# Pharmacokinetic-Pharmacodynamic Analysis on Inflammation Rat Model after Oral Administration of Huang Lian Jie Du Decoction

**DOI:** 10.1371/journal.pone.0156256

**Published:** 2016-06-09

**Authors:** Wei Ren, Ran Zuo, Yao-Nan Wang, Hong-Jie Wang, Jian Yang, Shao-Kun Xin, Ling-Yu Han, Hai-Yu Zhao, Shu-Yan Han, Bo Gao, Hao Hu, Yuan-Jia Hu, Bao-Lin Bian, Nan Si

**Affiliations:** 1 Institute of Chinese Materia Medica, China Academy of Chinese Medical Sciences, Beijing 100700, China; 2 Capital Medical University, Beijing 100069, China; 3 Li Kang Hospital, Beijing 102609, People’s Republic of China; 4 State Key Laboratory of Quality Research in Chinese Medicine, Institute of Chinese Medical Sciences, University of Macau, Macao SAR, China; 5 Key Laboratory of Carcinogenesis and Translational Research (Ministry of Education), Department of Integration of Chinese and WesternMedicine, Peking University School of Oncology, Beijing Cancer Hospital & Institute, Beijing 100142, PR China; 6 Anhui Jinchan Biochemistry Company Ltd., Huaibei 235000, China; Macau University of Science and Technology, MACAO

## Abstract

Huang-Lian-Jie-Du Decoction (HLJDD) is a classical Traditional Chinese Medicine (TCM) formula with heat-dissipating and detoxifying effects. It is used to treat inflammation-associated diseases. However, no systematic pharmacokinetic (PK) and pharmacodynamic (PD) data concerning the activity of HLJDD under inflammatory conditions is available to date. In the present study, the concentration-time profiles and the hepatic clearance rates (HCR) of 41 major components in rat plasma in response to the oral administration of a clinical dose of HLJDD were investigated by LC-QqQ-MS using a dynamic multiple reaction monitoring (DMRM) method. Additionally, the levels of 7 cytokines (CKs) in the plasma and the body temperature of rats were analyzed. Furthermore, a PK-PD model was established to describe the time course of the hemodynamic and anti-inflammatory effects of HLJDD. As one of the three major active constituents in HLJDD, iridoids were absorbed and eliminated more easily and quickly than alkaloids and flavonoids. Compared with the normal controls, the flavonoids, alkaloids and iridoids in inflamed rats exhibited consistently changing trends of PK behaviors, such as higher bioavailability, slower elimination, delays in reaching the maximum concentration (T_max_) and longer substantivity. The HCR of iridoids was different from that of alkaloids and flavonoids in inflamed rats. Furthermore, excellent pharmacodynamic effects of HLJDD were observed in inflamed rats. The levels of tumor necrosis factor-α (TNF-α), interleukin-6 (IL-6), IL-1β, IL-10, and macrophage inflammatory protein-2 (MIP-2) and body temperature significantly decreased after the administration of HLJDD. Based on PK-PD modeling with the three-phase synchronous characterization of time-concentration-effect, flavonoids exhibited one mechanism of action in the anti-inflammatory process, while iridoids and alkaloids showed another mechanism of action. Taken together, the results demonstrated that HLJDD may restrain inflammation synergistically via its major constituents (alkaloids, flavonoids and iridoids). A correlation between the exposure concentration of different types of compounds and their anti-inflammatory effects in the body was shown. This study provides a comprehensive understanding of the anti-inflammatory activity of HLJDD.

## Introduction

Huang-Lian-Jie-Du Decoction (HLJDD) is a classical Traditional Chinese Medicine (TCM) formula with heat-dissipating and detoxifying effects. It is used to treat inflammation-associated diseases. It is comprised of *Rhizoma coptidis*, *Radix scutellariae*, *Cortex phellodendri* and *Fructus gardenia*at a ratio of 3:2:2:3. HLJDD exerts various bioactivities on multiple types of tumors [1–5], arthritis [6–8], polymicrobial sepsis [9], cardiac damage [10], liver injuries [11], type II diabetes [12–14], and Alzheimer's disease [15]. These effects are closely associated with its anti-inflammatory activity, which is synergistically exhibited by the major ingredients in HLJDD [16].

Alkaloids, flavonoids and iridoids were reported to be the main bioactive constituents in HLJDD [16–18], and they individually act on specific targets in the inflammatory process [16, 19, 20]. Their preliminary pharmacokinetic behaviors have been studied in recent years [21–25]. However, only six constituents were examined in previous studies, which did not fully illustrate the complete plasma pharmacochemistry profile of HLJDD [21]. Additionally, it is difficult to obtain meaningful information on the efficacy and exposure concentration *in vivo* [23]. This unsatisfactory level of data can be attributed to imprecise experimental instruments, such as HPLC, which presents low sensitivity, poor selectivity and a limited ability to detect active components. In contrast, UHPLC combined with triple quadruple mass spectrometry (QqQ-MS) exhibits a faster analytical speed, a wider quantification range and higher sensitivity [26, 27]. These advanced features help facilitate the analysis of the formula and plasma profile of complex chemicals, such as TCM [18, 24].

Carrageenan (Ca) and lipopolysaccharide (LPS) have been used to induce inflammation *in vivo* [8, 20, 28, 29]. Some pure compounds isolated from HLJDD had positive effects on inflammation [2, 16, 30–34]. It is well known that separate pharmacodynamic assessments cannot fully describe the complete effect of HLJDD on inflammation. The pharmacokinetic-pharmacodynamic (PK-PD) method, which has been used extensively in drug screening, clinical trial design and the selection of dose regimens [35], is a feasible way to clarify the synergism of a formula’s multiple components [36]. In our previous study, the chemical profiling of the main constituents of HLJDD in rat plasma was comprehensively clarified, and 17 representative compounds in the extract of HLJDD were simultaneously quantified as quality control markers [18, 37]. Additionally, the distinctive metabolic processes of three types of representative components in HLJDD were clarified, and the *in vivo* metabolic network of HLJDD was illustrated [38]. These results laid a solid foundation for the PK-PD analysis of HLJDD in a pathological rat model.

In this pharmacokinetic study, we investigated the concentration-time profiles and the hepatic clearance rates (HCR) of 41 major components of HLJDD after the oral administration of a clinical dose in rats. LC-QqQ-MS in the dynamic multiple reaction monitoring (DMRM) mode was used for the PK analysis. Simultaneously, the body temperature and the levels of 7 cytokines (CKs), including TNF-α, IL-6, IL-1β, IFN-γ, IL-10, IL-13 andMIP-2, were estimated. Furthermore, a logistic transition mathematical model was established based on the PK and PD data to investigate the relationship between pharmacokinetic exposure to the active substances in HLJDD and the pharmacodynamic response. This study provided a comprehensive understanding of the anti-inflammatory activity of HLJDD.

## Materials and Methods

### Materials and Reagents

*Radix scutellariae*, *Rhizoma coptidis*, *Cortex phellodendri chinensis* and *Fructus gardenia* originated from the same batch as that was used in our previous reports [18, 37, 38]. Reference standards of berberine, baicalin, wogonin, oroxylin A and geniposide were isolated from HLJDD in our laboratory [39]. Magnolflorine was purchased from Beijing Saibaicao Co., Ltd. (Beijing, China). Wogonoside and oroxylin A-7-O-glucuronide were provided by Ze Lang Medical Technologies Co., Ltd. (Nanjing, China). Swertiamarin (IS1), corynoline (IS2) and icariin (IS3) were purchased from the National Institute for the Control of Pharmaceutical and Biological Products (Beijing, China) and employed as the internal standards for iridoids, alkaloids and flavonoids, respectively. Ascorbic acid (batch number: 20120214) was obtained from Sinopharm Chemical Reagent Co., Ltd. (Beijing, China). LPS (derived from *Escherichia coli* 0111: B4) and Ca were obtained from Sigma (St. Louis, MO, USA). HPLC-grade methanol and analytical-grade acetonitrile were purchased from Honeywell Burdick & Jackson (Swedesboro, NJ, USA). Formic acid was obtained from Thermo Fisher Scientific (Bremen, Germany),and ultra-pure water was purified by a Millipore system (Millipore, Billerica, MA, USA). Other chemicals and solvents were of analytical grade.

AimPlex^™^ assay kits, including an AimPlex^®^ Rat custom 7-plex kit (cat number: T311C07) and an AimPlex^®^ Mouse/Rat Basic kit (cat number: R200201), were provided by Beijing Quantobio Biotechnology Co.,Ltd. (Beijing, China).

### Plant Extraction and Preparation of Solutions

Four samples of dried and crushed plant material were homogenized at a ratio of 3:2:2:3 (*Coptidis rhizoma*:*Scutellariae radix*:*Phellodendri chinensis cortex*:*Fructus gardeniae*) and decocted twice with boiling water (1:10, w/v) for 2 h. The aqueous extract was concentrated to a constant weight on a rotary vacuum evaporator at 80°C and crushed into powder before the experiment. HLJDD extract powder (HLJDD-EP), Ca and LPS were individually dissolved in saline solution via ultrasonication at a concentration of 35.0 mg/mL for HLJDD-EP, 25.0 mg/mL for Ca and 50.0 μg/mL for LPS. Eight reference standards (geniposide, magnolflorine, baicalin, berberine, oroxylin A-7-O-glucuronide, wogonoside, wogonin and oroxylin A) were dissolved in methanol and diluted to a series of concentrations. An internal standard stock solution (containing swertiamarin, corynoline and icariin) was also prepared with methanol.

### Surgical Procedure and Collection of Biosamples

Sprague-Dawley rats (male, 200±20 g), provided by Cisco North Biotechnology Co., Ltd.(Beijing, China), were maintained in polypropylene cages with constant access to rodent chow (Nanjing, China) and water in an environmentally controlled room (12 h light cycle) at 20±1°C and 50±10% relative humidity. The rats were acclimatized to the facilities for 5 days and screened via the continuous determination of the rectal temperature for two days. Rats with temperatures over 38°C and those that exhibited a difference between two measured temperatures greater than or equal to 0.5°C were excluded from the experiment. Rats in the inflamed groups previously received intraperitoneal injections of Ca (0.1 mL/100 g) and were fasted with free access to water for 16 h, followed by intravenous injections of LPS (0.1 mL/100 g) to induce inflammation. The control group G1 was injected with the same volume of physiological saline. The ethics committees of Cisco North Biotechnology Co., Ltd. (Beijing, China) and the China Academy of Chinese Medical Sciences (Beijing, China) approved the experimental protocol. The ethical approval number was BJ-2014-0125-01.

For the PK-PD analysis, cannulas were implanted in each rat’s jugular vein (n = 22). To study the hepatic first-pass effect (HFPE), the jugular vein and the pyloric vein of each rat (n = 5) were cannulated according to the method described by Hye J. Chung *et al*. with slight modifications [40]. Instead of the portal vein, the pyloric vein was cannulated to minimize the interference of blood flow in the portal vein [41]. The surgery was performed under anesthesia (3% chloral hydrate solution, intraperitoneal injection; 3.5 mL/kg) 5 days prior to the experiment. Rats with jugular vein intubation surgery were divided into four groups: a control group with HLJDD (G1, n = 6), aninflammatory group with HLJDD (G2, n = 6), aninflammatory group with physiological saline (G3, n = 5) and another inflammatory group with HLJDD (G4, n = 5). G1 and G2 were used for the pharmacokinetic study, while G3 and G4 were used for the pharmacodynamic study. Rats with jugular and pyloric vein cannulation for HFPE analysis were assigned to another inflammatory group with HLJDD (G5, n = 5).

The prepared HLJDD-EP solution was administered to the rats (G1, G2, G4 and G5) at 2 mL/100g body weight (crude material content: 3.5 g/kg) by oral gavage, and the G3 group was given the same volume of physiological saline. In the G1 and G2 groups, blood specimens (300 μL) were obtained before dosing and subsequently at 5 min, 10 min, 30 min, 1, 2, 4, 6, 8, 12, 24, 36 and 48 h after the oral administration of HLJDD-EP solution. In the G3 and G4 groups, blood samples (300 μL) were collected prior to dosing and followed at 10 min, 30 min, 1, 2, 4, 8, 24 and 48 h after drug administration. The rectal temperature of rats in the G3 and G4 groups was immediately monitored after each collection of blood. For the HFPE assay, blood specimens (150 μL) from both the jugular vein and the pyloric vein of the G5 rats were simultaneously taken before dosing and subsequently at 5 min, 10 min, 30 min, 1, 2, 4, 6, 8, 12, 24, 36 and 48 h after oral administration. Samples were collected in heparinized Eppendorf tubes and centrifuged at 3000 rpm for 15 min. The plasma was stored at -80°C until assay.

### Determination of the 41 Components of HLJDD in Rat Plasma

For the pharmacokinetic investigation and HFPE analysis, 100 μL aliquots of plasma were mixed with 5 μL of ascorbic acid (dissolved in physiological saline, w/v: 1 g/100 mL) and 200 μL of methanol (containing an internal standard stock solution with 80.0 ng/mL swertiamarin, 1.5 ng/mL corynoline and 33.0 ng/mL icariin), followed by vortexing and centrifuging at 15000 rpm for 20 min to remove the precipitate. The supernatant was dried with nitrogen at 40°C. The residue was re-dissolved in 100 μL of methanol and centrifuged at 15000 rpm for 10 min. The supernatant was analyzed using Agilent 1290 ultra-high performance liquid chromatography coupled with an Agilent 6490 Triple Quadrupole Mass Spectrometer (Agilent Technologies, Palo Alto, CA, USA).

Liquid chromatographic separations of the analytes were performed using a Thermo Scientific Hypersil GOLD column (50 mm× 2.1 mm, 1.9 μm). The mobile phase consisted of acetonitrile (solvent A) and 0.1% formic acid in water (solvent B). The gradient elution was as follows: 0–3 min, linear from 5% to 10% A; 3–10 min, linear from 10% to 23% A; 10–15 min, linear from 23% to 50% A; 15–16 min, linear from 50% to 100% A; 16–17 min, held at 100% A; 17–18 min, linear from 100% to 5% A; and 18–20 min, held at 5% A for equilibration of the column. The flow rate was 0.3 mL/min. The injection volume was 2 μL. The column temperature was kept at 35°C, and the sampler temperature was set at 4°C. The optimized ESI source parameters were as follows: gas temperature, 200°C; gas flow, 14 L/min; nebulizer pressure, 20 psi; sheath gas temperature, 400°C; and sheath gas flow, 11 L/min. Analytes were quantitated by monitoring the precursor-product combination in the DMRM mode using ion polarity switching (flavonoids and alkaloids were determined in the positive mode, while iridoids were detected in the negative mode). To ensure the desired abundance of each compound, the CE values and other parameters were optimized and illustrated as follows: cycle time, 300 ms; positive capillary voltage, 3000 v; negative capillary voltage, 2000 v; positive nozzle voltage, 1500 v; negative nozzle voltage, 1000 v; Delta EMV(+), 200 v; Delta EMV(-), 200 v. The optimized mass transition ion pairs (*m/z*) for analytes and the detection of the conditions of the compounds are shown in S1 Table.

The current UHPLC-QqQ-MS method was validated for its linearity, intra-day and inter-day precision, accuracy, stability, extraction recovery and matrix effect. The typical chromatograms of plasma samples showed an ideal signal response, excellent resolution, clear exhibition of shapes and short run time (S1 Fig). The regression equations, correlation coefficients, test ranges and limits of quantification (LOQ) are shown in S2 Table. All the calibration curves showed an excellent correlation between the ratio of peak area and concentrations for each compound within the test ranges. The intra- and inter-day precision of these analytes (relative error, RE) were less than -17.9% and 14.8%, respectively (S3 Table). The 8 analytes in plasma were stable (S4 Table) under the following conditions: 4 h at room temperature, 15 days of storage at -80°C, 3 freeze-thaw cycles at -80°C and 48 h in the autosampler. The extraction recoveries of the 8 standards (S5 Table) were all within the acceptable ranges (from 82.41% to 105.98%), except for baicalin (from 68.52% to 80.20%), and all of the results showed good accuracy and precision. The low extraction recovery of baicalin was in accordance with the published results that its mean recovery in rat plasma ranged from 70.51% to 75.28% [42]. Most of the average matrix effect values of the analytes at 3 quality control (QC) concentrations ranged from 80% and 120%, except for baicalin (ranged from 68.13% to 75.73%), which indicated that no co-eluting unseen compounds significantly influenced the ionization of the analytes.

### Simultaneous Measurements of 7 Cytokines in Rat Plasma

The measurement of 7 cytokines, including IFN-γ, IL-6, IL-1β, MIP-2, TNF-α, IL-13 and IL-10in rat plasma was conducted using a commercially available rat flow cytomix basic kit (BD Biosciences). All of the procedures were performed by the same operator according to the manufacturer’s protocol. Standard curves for each biomarker were generated with 7 serial concentrations following the manufacturer’s instructions.

### Data Analysis

Pharmacokinetic parameters of the determined compounds were processed by the non-compartmental method using Phoenix Winnonlin software (Pharsight Corporation, USA). Linear trapezoidal integration was used to calculate areas under the concentration-time curves (AUC) and areas under the first moment curves (AUMC). The mean residence time (MRT) was determined as AUMC/AUC. The plasma half-life (HL_λz_), maximum plasma concentration (C_max_), the time to reach C_max_ (T_max_) and other parameters following the administration of HLJDD were determined from the observed data. The HFPE was defined as HCR: HCR% = 100×(AUC_0−t_ in the pyloric vein-AUC_0−t_ in the jugular vein)/AUC_0−t_ in the pyloric vein. Concentrations of cytokines were analyzed by cytometric bead array software (FCAP Array version 3.0, BD Bioscience). All the data were expressed as the mean±standard deviation (SD). A *p* value less than 0.05 was considered statistically significant.

Based on the PK and PD data, a PK-PD fitted mathematic model was established to investigate the relationship between the active substances in HLJDD and the pharmacodynamic indexes. After continuity correction with a logistic steady transformation, the empirical distribution function of data for each time point was calculated. To form virtual sample paths, the bootstrap re-sampling method was used for random data extraction from each time point. Thus, the pharmacokinetic and pharmacodynamic measurement of one rat was emulated. In the present study, the number of re-samplings was set as M = 200. The logistic transformation formula was as follows:

$$R(t)=\frac{1}{1+\text{exp}\left\{\beta (x-\alpha )\right\}}$$

α and β represent the positional parameters and scale parameters for each variable, respectively.

The cumulative effect of each time point was selected as the characteristic index in the analysis. The cumulative index of the control group was utilized as a background to correct the baseline drift. The polynomial interpolation method was used to obtain each value of each time point along the virtual paths. Furthermore, the average effect of the pharmacokinetic indexes on the pharmacodynamic indexes was investigated by establishing a dynamic model of stochastic differential equations (defined below):

dY(t)/dX(t)=A(t)+ε(t)

X(t) and Y(t) represent the cumulative effect of the various indexes, and A(t) represents the change rate of the pharmacodynamic indexes with respect to the pharmacokinetic indexes.

The cubic spline method was used to calculate the values of the partial derivative for each virtual efficacy path in terms of the aforementioned 8 components. As a result, the ingredient and effect index of every virtual path at any point in time produced a series of derivative values (M = 200). Then, the density estimation of the partial derivative from the effectiveness index of each ingredient at every time point was obtained by kernel density estimation. If the efficacy of an ingredient was not significant, the probability density of the derivative approached zero; otherwise, it moved to positive or negative areas. Based on their average variation rate, the relationship between the pharmacokinetic and pharmacodynamic indexes was deduced.

## Results

### Measurement of 41 Components in Rat Plasma

**Fig 1:**
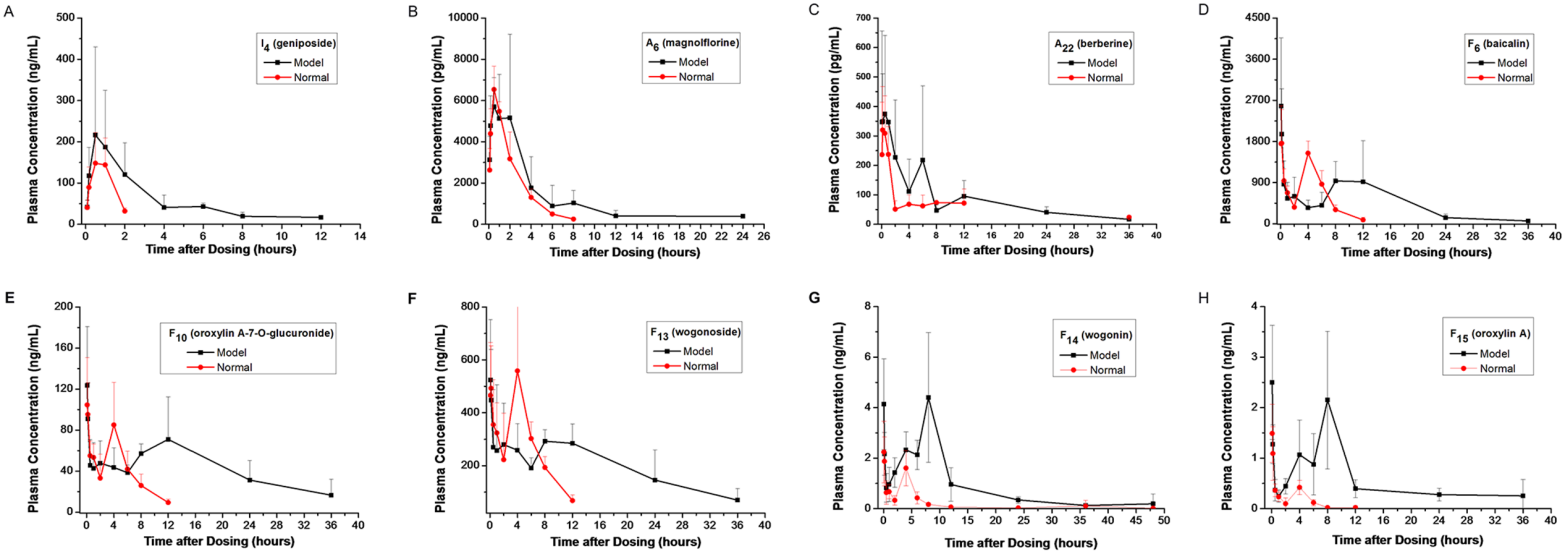
The plasma concentration-time curves of geniposide, magnolflorine, berberine, baicalin, oroxylin A-7-O-glucuronide, wogonoside, wogonin and oroxylin A in normal and inflamed rats.

The validated method was successfully applied to the pharmacokinetic and HFPE analysis of 41 compounds in rat plasma. The plasma concentration-time curves (n = 6) of geniposide, magnolflorine, berberine, baicalin, oroxylin A-7-O-glucuronide, wogonoside, wogonin and oroxylin A in normal rats are shown in Fig 1, and their estimated pharmacokinetic parameters are presented in Table 1. The peak area ratio-time curves (n = 6) of the other 33 ingredients are shown in S2 Fig. The results demonstrated that iridoids were absorbed and eliminated more easily and quickly than alkaloids and flavonoids. The T_max_ and MRT_last_ of geniposide were 0.7±0.3 h and 0.9±0.1 h, respectively. The pharmacokinetic behaviors of aporphine alkaloids were different from those of protoberberine alkaloids, which may have been caused by differences in their absorption in the gastrointestinal tract. Flavonoids exhibited similar T_max_ and MRT in rat plasma. All of the flavonoids exhibited a bimodal phenomenon in rats, with C_max1_ at 5–10 min and C_max2_ at 8–12 h, which was consistent with previously published reports [22, 43].

**Table 1:** The pharmacokinetic parameters of geniposide, magnolflorine, baicalin, berberine, oroxylin A-7-O-glucuronide, wogonoside, wogonin and oroxylin A in normal and inflamed rats.

PK parameters	Group	baicalin	oroxylin A-7-O-glucuronide	wogonoside	geniposide	magnolflorine	berberine	wogonin	oroxylin A
**Dose (g/kg)**	B/M	0.1246	0.0236	0.0910	0.0881	0.0024	0.0296	0.0063	0.0025
**HL**_**λz**_ **(h)**	B	1.8±0.4	3.2±0.9	3.0±1.0	0.6±0.3	1.6±0.4	6.3±3.5	3.5±1.3	1.7±0.9
	M	4.7±1.1	20.1±6.1	12.2±5.9	1.6±0.7	4.3±2.2	9.1±2.5	7.1±2.7	16.3±8.8
**Tmax1 (h)**	B	0.09±0.03	0.09±0.03	0.11±0.04	0.7±0.3	0.6±0.2	0.6±0.4	0.09±0.03	0.08±0.0
	M	0.1±0.04	0.09±0.03	0.16±0.08	0.8±0.3	0.6±0.3	1.0±0.6	0.08±0.0	0.08±0.0
**Tmax2 (h)**	B	4.3±0.8	4.0±0.0	4.3±0.8	-	-	8.0±2.2	4.0±0.0	4.0±0.0
	M	9.3±2.1	9.6±2.2	8.7±3.0	-	-	10.8±2.7	7.3±2.7	7.0±3.0
**Cmax1 (ng/ml)**	B	1828.8±737.4	106.9±43.8	492.9±152.9	171.6±70.6	6.5±1.1	0.4±0.1	2.2±1.2	1.5±0.6
	M	2420.9±922.5	108.7±47.6	571.9±183.4	223.2±43.7	6.0±1.7	0.5±0.2	3.3±0.7	2.5±1.1
**Cmax2 (ng/ml)**	B	1580.1±202.4	85.8±40.3	517.7±173.8	-	-	0.1±0.03	1.6±0.7	0.4±0.1
	M	1000.4±298.1	65.5±18.2	323.9±52.5	-	-	0.3±0.3	4.3±1.8	2.3±0.8
**AUC**_**last**_ (h*ng/ml)	B	7881.1±774.7	440.7±106.9	3302.3±975.1	216.8±86.2	16.0±2.0	1.3±0.5	7.2±2.6	2.3±0.5
	M	15737.7±9300.3	1209.8±606.4	6870.4±1786.5	637.6±323.1	24.9±14.6	2.7±0.8	38.7±8.1	21.1±4.1
**AUC**_**inf**_ (h*ng/ml)	B	8129.0±906.2	486.8±96.9	3615.4±872.3	239.9±82.1	17.0±2.0	1.8±0.6	7.4±2.6	2.4±0.5
	M	16842.2±9089.7	2157.4±785.0	8380.9±1944.5	695.7±362.8	29.3±13.8	3.4±0.7	39.2±8.0	24.6±6.1
**AUC**_**%Extrap**_ **(%)**	B	2.9±2.3	10.3±6.8	9.6±6.6	11.5±1.5	6.2±2.4	25.9±15.5	2.7±2.3	5.3±3.4
	M	2.7±0.5	46.2±12.3	17.3±13.8	8.9±2.9	17.6±8.0	10.7±7.0	1.3±0.7	13.4±8.6
**V**_**z**_**/F (L/kg)**	B	39.6±7.1	247.8±140.4	118.7±58.0	235.1±94.2	339.5±97.3	145349.8±40480.7	4013.3±1353.8	2496.7±1078.9
	M	58.8±25.2	322.4±22.0	188.6±72.3	372.2±86.7	607.8±396.5	118054.1±38377.6	1706.5±666.9	2822.5±507.0
**CL/F (L/h/kg)**	B	15.5±1.7	50.7±13.5	26.5±7.0	411.5±160.9	142.6±15.9	18437.4±7250.1	763.9±114.0	1089.6±241.5
	M	8.7±3.2	12.1±4.2	11.4±2.7	225.4±108.6	923.7±274.2	9105.5±2316.3	164.6±28.8	107.4±27.0
**AUMC**_**last**_ (h*h*ng/ml)	B	34124.1±4967.9	1935.2±487.3	15357.0±3545.6	192.5±74.8	29.5±5.8	8.9±8.6	31.8±14.3	7.1±2.7
	M	175077.1±103375.1	18194.0±6706.5	92756.1±44786.8	1231.9±677.2	97.9±55.4	22.6±8.6	442.5±232.9	365.4±192.9
**MRT**_**last**_ **(h)**	B	4.3±0.3	4.4±0.5	4.7±0.5	0.9±0.1	1.8±0.1	5.7±3.5	4.3±0.8	3.0±0.6
	M	10.1±2.3	12.5±2.3	12.9±3.0	1.9±0.8	4.1±1.9	8.4±3.1	11.0±3.5	16.5±6.2

B, normal group with HLJDD; M, inflamed group with HLJDD

Compared to the controls, the pharmacokinetic behaviors, including HL_λz_, T_max_ and AUCs of most flavonoids, alkaloids and iridoids significantly increased in inflamed rats after the oral administration of the HLJDD extract. It was observed that the AUC_last_ values of geniposide, magnolflorine, berberine, baicalin and wogonin in inflamed rats were 637.6, 24.9, 2.7, 15737.7 and 38.7 h*ng/ml, respectively, while those in normal rats were 216.8, 16.0, 1.3, 7881.1 and 7.2 h*ng/ml, respectively. The mean AUC_last_ values of geniposide, magnolflorine, berberine, baicalin and wogonin plasma concentrations were 2.9-, 1.6-, 2.1-, 2.0- and 5.4-fold larger in the inflamed group than those in the control group.

**Fig 2:**
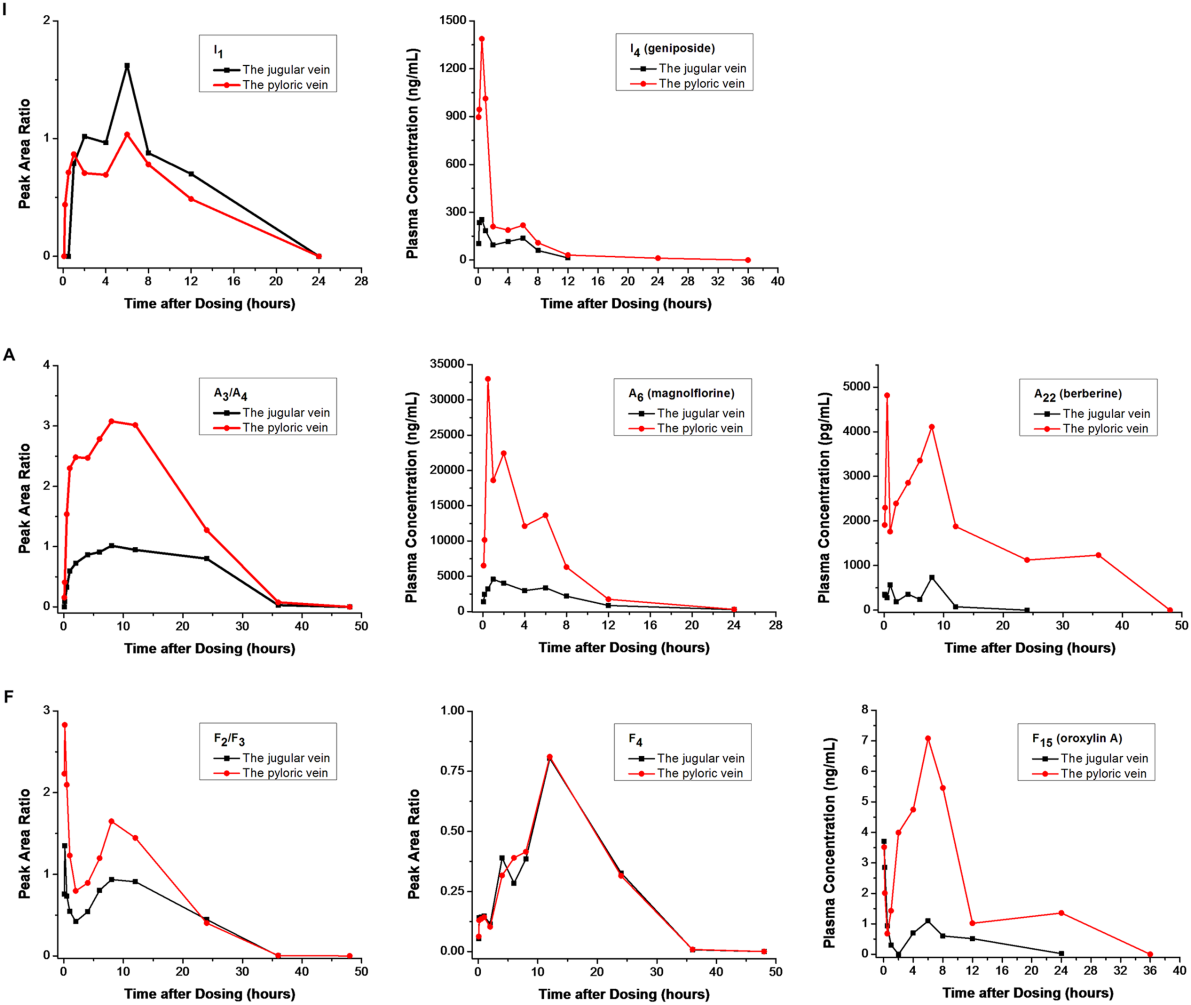
The representative concentration and peak area ratio-time curves of the typical components in the plasma from the jugular vein and the pyloric vein of the inflamed rats (I, iridoids; A, alkaloids; F, flavonoids).

**Fig 3:**
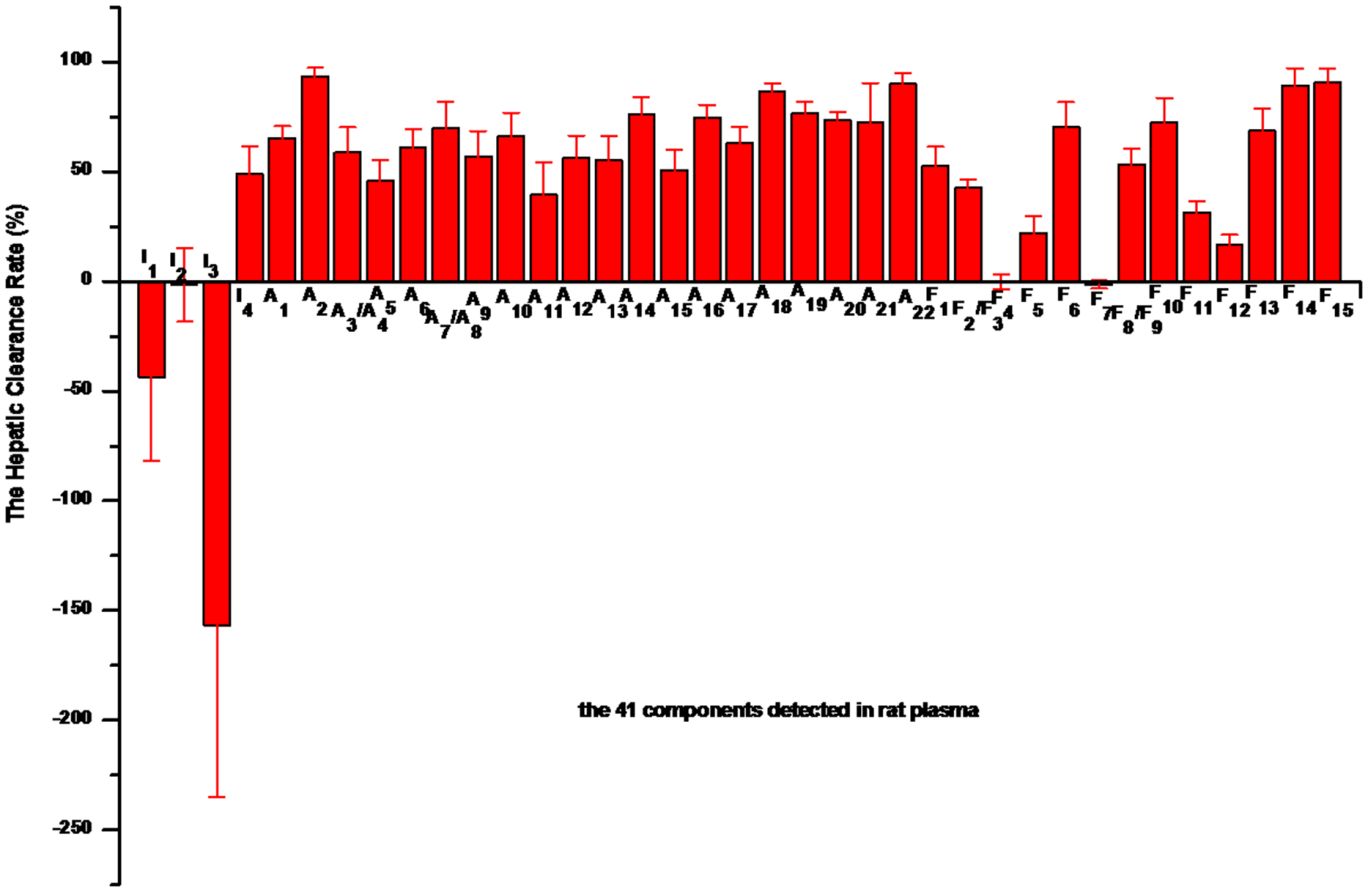
The hepatic clearance rates of the 41 constituents in inflamed rats.

According to the obtained AUC_0−t_ of the 41 compounds in the plasma obtained from the jugular vein and the pyloric vein of the inflamed rats (Fig 2), the HCR was calculated (Fig 3). The HCR values of I_1_and I_3_ were -43.7% and -156.6%, respectively, due to the cross-metabolism of iridoids. Specifically, some iridoids were metabolized into I_1_ and I_3_ in the liver. It is worth noting that the HCR values of I_2_, F_4_ and F_7_ were approximately zero, indicating that these compounds exhibited good bioavailability after oral administration. In comparison, all of the alkaloids and most of the flavonoids exhibited a relatively higher HCR value.

### Pharmacodynamic Analysis of HLJDD

**Fig 4:**
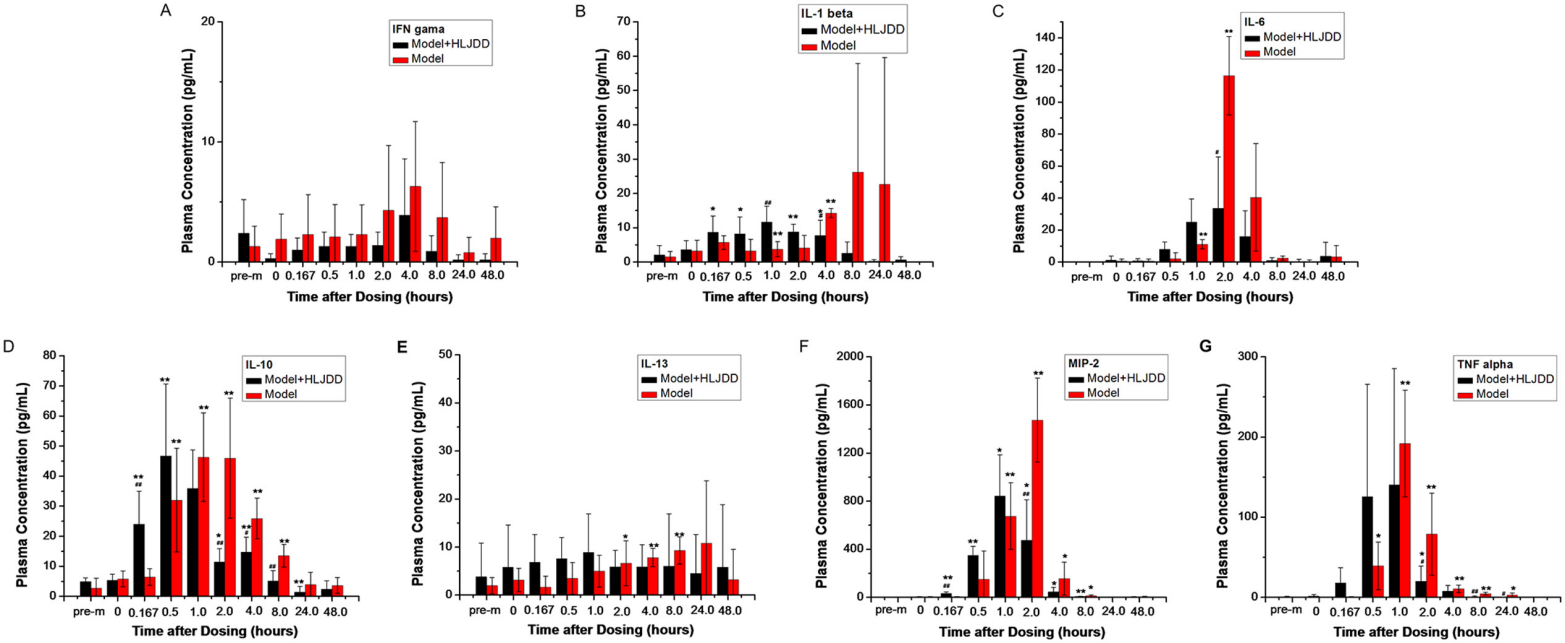
The levels of the 7 cytokines (IL-6, IL-1β, MIP-2, IFN-γ, TNFα, IL-13 and IL-10) in G3 and G4 rats (pre-m, pre-modeling of inflammation; *P<0.05,**P<0.01, before and after modeling; #P<0.05, ##P<0.01, differences between G3 and G4).

Significant differences in body temperature were found in the inflamed rats before and after modeling. All of the cytokines except IFN-γ increased at a certain time point between normal and model rats (Fig 4).The results suggested that CKs, including IL-6, IL-1β, MIP-2, TNF-α, IL-13 and IL-10, were closely associated with the inflammation induced by LPS and Ca. These findings are consistent with the published literature [44]. Furthermore, levels of IL-6, IL-1β, MIP-2, TNF-α, and IL-10and body temperature were down-regulated after the administration of HLJDD. Specifically, HLJDD may significantly reduce the expression of MIP-2, TNF-α and IL-6 at 2 h, IL-1β at 4 h and IL-10 at 2–8 h in the plasma of inflamed rats and down-regulate the body temperature 8 h after the oral administration of HLJDD (Table 2). The results indicated that HLJDD may prevent an excessive inflammatory response under acute inflammatory conditions.

**Table 2:** Measurement of the body temperature of G3 and G4 rats (n = 5).

Time	Body temperature of rats in G3 (°C)	Body temperature of rats in G4 (°C)
**Pre-modeling of inflammation**	36.45±0.45	36.14±0.29
**After modeling of inflammation**		
0 min	36.83±0.09	36.68±0.49
10 min	36.84±0.19	36.39±0.73
30 min	36.50±0.18	36.36±0.58
1 h	36.49±0.12	36.53±0.21*
2 h	36.48±0.25	36.41±0.14
4 h	37.60±0.30**	37.77±0.58**
8 h	37.68±0.19**	36.87±0.51* ##
24 h	35.87±0.50	36.08±0.06
48 h	36.07±0.33	35.98±0.11

*P<0.05,

**P<0.01 (before and after modeling);

^#^P<0.05,

^##^P<0.01 (differences between G3 and G4)

### Pharmacokinetic-Pharmacodynamic Analysis

**Fig 5:**
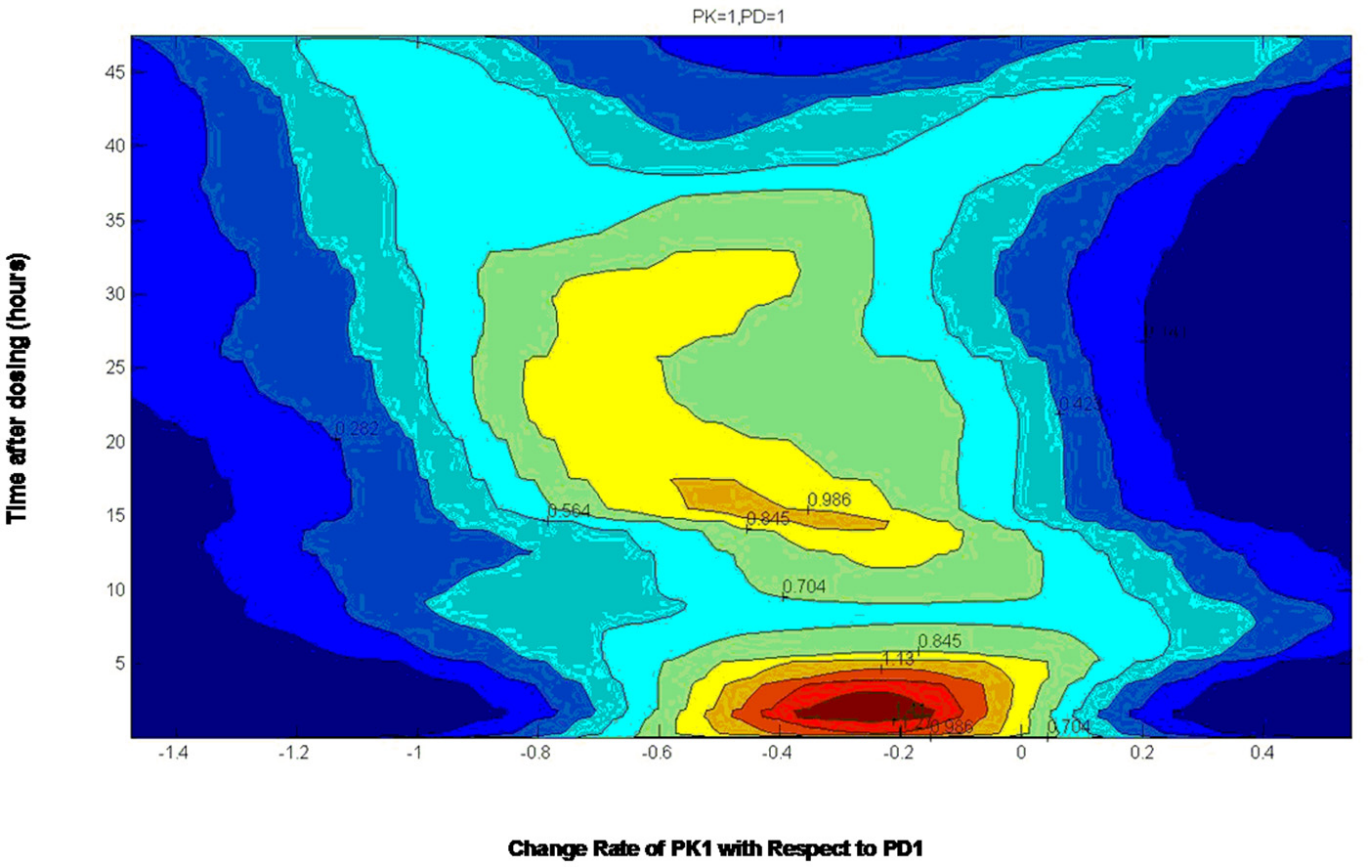
The average change rate of PK1 (baicalin) with respect to PD1 (IL-6). The brightness of the colors represents the probability density of the related indexes.

**Fig 6:**
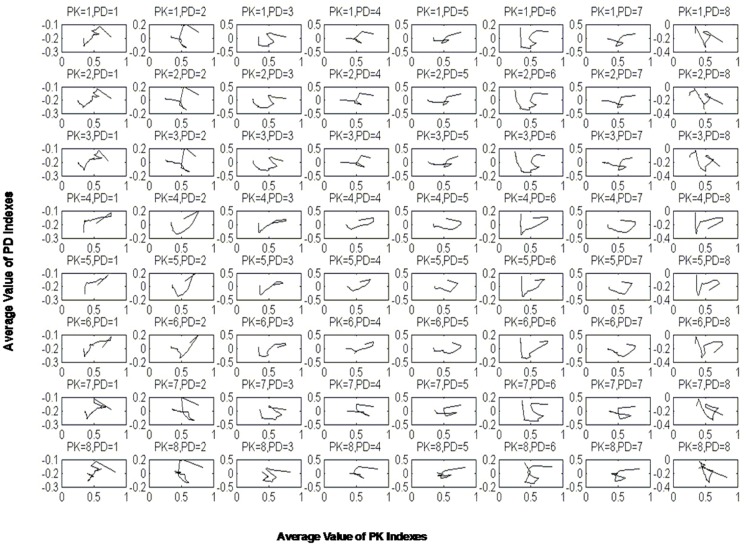
The relationship between pharmacokinetics and pharmacodynamics. Each subgraphic represents the change in the trend of a PD index along with a PK index; PK indexes 1–8 represent baicalin, oroxylin A-7-O-glucuronide, wogonoside, geniposide, magnolflorine, berberine, wogonin and oroxylin A, respectively; PD indexes 1–8 represent IL-6, IFN-γ, IL-1β, MIP-2, TNF-α, IL-13, IL-10 and the body temperature of rats, respectively.

First, equipotential curves were fabricated based on the probability density matrix to clearly express the change in the rates of pharmacodynamic indexes to pharmacokinetic indexes. For example, from PK1 (baicalin) to PD1 (IL-6) (Fig 5), the average rate of change exhibited greater density, with a red color in the range of -0.4 to -0.1 (negative value), indicating the inhibitory effect of PK1 on PD1. Furthermore, the relationship between pharmacokinetics and pharmacodynamics was depicted based on the PK-PD analysis. As shown in Fig 6, the relationship between the PK indexes and PD indexes was not simply linearly related, demonstrating a complex interrelationship and the delayed effect of PK indexes. Interestingly, 5 PK indexes of flavonoids, including PK1 (baicalin), PK2 (oroxylin A-7-O-glucuronide), PK3 (wogonoside), PK7 (wogonin) and PK8 (oroxylin A), presented similar mechanisms of regulating the PD indexes. In comparison, the PK indexes of iridoid (PK4, geniposide) and alkaloids, including PK5 (magnolflorine) and PK6 (berberine), may employ the other mechanism. These results provide a new perspective for elucidating the active substances of HLJDD and their mechanism of treating inflammation.

## Discussion

In the pharmacokinetic study, the flavonoids demonstrated a bimodal phenomenon. The results, especially those for baicalin and wogonoside, were in accordance with those of a previous report [22]. Baicalin was first hydrolyzed into baicalein by β-glucuronidase in the intestinal tract. Then, baicalein was absorbed and metabolized to baicalin in the liver. The baicalin formed in the liver was excreted into the duodenum with bile, which was re-absorbed from the intestine via the enterohepatic circulation [45–47]. Additionally, baicalin was excreted into the gut lumen through multi-drug resistance-associated protein2 (MRP2) after transformation in the intestinal mucosa [48]. Furthermore, protein combination [49] and the absorption of flavonoids at two sites have also been associated with the bimodal phenomenon of flavonoids. For example, it was reported that the first absorption site of baicalin was the upper intestine and the second site was the colon [50]. All of the above findings may have accounted for the double peak phenomenon of flavonoids in rats after the oral administration of HLJDD.

In the present study, the pharmacokinetic parameters of the 41 compounds in inflamed rats were different from those in normal rats after the oral administration of HLJDD. This difference may have resulted from the influence of the pathological status [22, 43, 51], which led to alterations in the function of drug metabolizing enzymes, such as cytochrome P450, β-glucuronidase and UDP-glucuronosyl transferase, together with the expression of drug transporters like P-glycoprotein [52, 53]. In addition, the permeability of the vascular and intestinal mucosal barrier was enhanced in inflamed rats. Thus, the absorption of drugs increased and elimination became slower.

Based on the difference in the blood concentrations between the input and output of the liver, the local hepatic metabolism of HLJDD was evaluated by HCR experiments. According to the results, all of the alkaloids and most of the flavonoids exhibited a relatively higher HCR than iridoids. The HCR values of I_1_ and I_3_ were -43.7% and -156.6%, respectively, while that of I_2_ was close to zero. Many oral drugs are metabolized in the digestive tract and liver before they travel to the target organs [54]. The low bioavailability of oral drugs is due to intestinal bioconversion [55] or the liver and intestinal first-pass effect [40, 56–58]. The HCR and pharmacokinetic behaviors of the main ingredients in HLJDD provide beneficial guidance for its clinical use.

HLJDD was first recorded in the book "Wai Tai Mi Yao" by Wang Tao in the Tang Dynasty. Its four herbs have been officially listed in the Chinese Pharmacopoeia. HLJDD is commonly used in the treatment of inflammation-related diseases, such as systemic inflammatory response syndrome [59], acute gouty arthritis [60] and ulcerative colitis [61]. According to pharmacological researches, the anti-inflammatory activity of HLJDD plays a significant role in cecal ligation, puncture-induced liver and lung injury [9], type II diabetes [13, 62] and cardiac damage induced by metabolic disorder in rats [10]. Both the pure compounds [2, 16, 30–34] and the extracts in HLJDD [63]exerted positive effects on inflammation. Three major types of active substances (iridoids, flavonoids and alkaloids) present a synergistic anti-inflammatory effect [16]. However, the relationship between the PK behaviors of various types of constituents absorbed in the plasma and their effects is still not clear. The development of advanced instruments and the time-concentration-effect analysis of HLJDD is necessary. Therefore, our study sought to elucidate the anti-inflammatory effect of HLJDD and aimed to clarify the time-concentration-effect of HLJDD in an animal model of inflammation. It is generally known that inflammatory CKs can be used for the evaluation of inflammation and prognosis [28, 44, 64]. Meanwhile, HLJDD may block the release of inflammatory mediators [65] and reduce the levels of the inflammation-related cytokines TNF-α, IFN-γ, IL-1β, IL-6 and IL-17 in the inflammation model [6, 9, 20, 66]. HLJDD may inhibit Th17 activation, reverse the shift from the Th1 to Th2 response, promote the Th1/Th2 balance toward Th1 predominance [9] and inhibit the activation of NF-κB [10, 30]. Our research demonstrated that HLJDD may significantly reduce the expression of MIP-2, TNF-α and IL-6 at 2 h, IL-1β at 4 h and IL-10 at 2–8 h in inflamed rats and down-regulate the body temperature at 8 h to achieve its anti-inflammatory effect. In logistic transition mathematical analysis, the flavonoids displayed a similar mechanism of action, while iridoids and alkaloids employed another mechanism. These results supported the consensus that the pharmacological activities of the same type of compounds were similar to each other [67]. There is a growing consensus that TCM is a complex system, and a single component is not sufficient to characterize its pharmacokinetic process *in vivo*. The active prototypes of TCM and their metabolites *in vivo* constitute the material foundation of medicinal effectiveness. Based on our previous studies [37, 38], a total of 41 constituents, including flavonoids, alkaloids and iridoids, were detected in rat plasma. Among the 41 compounds, some were prototypes and the others were metabolites. Thus, they were chosen as pharmacokinetic markers to comprehensively illustrate the pharmacokinetic behavior of HLJDD *in vivo* and subsequently investigate the correlation of different types of compounds (flavonoids, alkaloids, iridoids, prototypes and metabolites) between the exposure dose and their anti-inflammatory effects in rats. The present study provided meaningful evidence to facilitate the understanding of the anti-inflammatory activity of HLJDD as an integrated system.

## Conclusions

In summary, the pharmacokinetic behavior and HCR of the main ingredients in HLJDD were investigated in this paper. A time-concentration-effect PK-PD analysis of HLJDD in an acute inflammation model was developed. The pharmacokinetic parameters (especially AUCs and C_max_) in inflamed rats were significantly increased compared to those in normal rats. The HCR of the major components of HLJDD were approximately 50%, demonstrating a moderate liver first-pass effect on HLJDD *in vivo*. This provides beneficial guidance for its clinical use. The mechanism by which HLJDD synergistically treats inflammation with its three major types of compounds (alkaloids, flavonoids and iridoids) was successfully verified. In addition, a visual PK-PD mathematical model was established to verify the accuracy of the results. This study provided corroborative evidence and a comprehensive understanding of the anti-inflammatory effects of HLJDD for clinical applications. Research on TCM formulas and the relationship between responses and exposure doses will facilitate the development of future clinical applications of these compounds.

## Supporting Information

S1 Fig The extracted chromatograms of 41 constituents in samples. Samples from left to right are rat plasma after oral administration of HLJDD, extract of HLJDD and blank plasma of rats.
(PDF)

S2 Fig The peak area ratio-time curves of the other 33 ingredients in normal and inflammation rats. (PDF)

S1 Table The selected detecting ions, collision energy (CE) and detecting conditions of the analytes. (DOCX)

S2 Table Calibration curves and LODs of geniposide, magnolflorine, baicalin, berberine, oroxylin A-7-O-glucuronide, wogonoside, wogonin and oroxylin A. (DOCX)

S3 Table Precision and accuracy of geniposide, magnolflorine, baicalin, berberine, oroxylin A-7-O-glucuronide, wogonoside, wogonin and oroxylin A. (DOCX)

S4 Table Stability of geniposide, magnolflorine, baicalin, berberine, oroxylin A-7-O-glucuronide, wogonoside, wogonin and oroxylin A in rat plasma (n = 3). (DOCX)

S5 Table Extraction recovery and matrix effect of geniposide, magnolflorine, baicalin, berberine, oroxylin A-7-O-glucuronide, wogonoside, wogonin and oroxylin A in rat plasma (n = 6). (DOCX)

## References

[1] Lin LT, Wu SJ, Lin CC. The Anticancer properties and apoptosis-inducing mechanisms of cinnamaldehyde and the herbal prescription Huang-Lian-Jie-Du-Tang (Huang Lian Jie Du Tang) in human hepatoma cells. J Tradit Complement Med. 2013;3(4):227–33. 10.4103/2225-4110.119732 24716182 PMC3924998

[2] Ma Z, Otsuyama K, Liu SQ, Abroun S, Ishikawa H, Tsuyama N, et al Baicalein, a component of Scutellaria radix from Huang-Lian-Jie-Du-Tang (HLJDT), leads to suppression of proliferation and induction of apoptosis in human myeloma cells. Blood. 2005;105(8):3312–8. 15626742 10.1182/blood-2004-10-3915

[3] Sun J, Wen QH, Song Y, Li X, Jin J, Ma JS, et al Study on antitumor activities of huanglian jiedu decoction. China J Chin materia medica. 2006;31(17):1461–3. 17087092

[4] Sun J, Wen QH, Li X, Song Y, Jin J, Ma JS, et al Comparison between antitumor effect and chemical constituents of Huanglian Jiedu decoction and that of serum containing Huanglian Jiedu decoction. China J Chin materia medica. 2006;31(18):1526–9. 17144472

[5] Wang N, Feng Y, Tan HY, Cheung F, Hong M, Lao L, et al Inhibition of eukaryotic elongation factor-2 confers to tumor suppression by a herbal formulation Huanglian-Jiedu decoction in human hepatocellular carcinoma. J Ethnopharmacol. 2015;164:309–18. 10.1016/j.jep.2015.02.025 25700642

[6] Zhang H, Fu P, Ke B, Wang S, Li M, Han L, et al Metabolomic analysis of biochemical changes in the plasma and urine of collagen-induced arthritis in rats after treatment with Huang-Lian-Jie-Du-Tang. J Ethnopharmacol. 2014;154(1):55–64. 10.1016/j.jep.2014.03.007 24709313

[7] Yue R, Zhao L, Hu Y, Jiang P, Wang S, Xiang L, et al Rapid-resolution liquid chromatography TOF-MS for urine metabolomic analysis of collagen-induced arthritis in rats and its applications. J Ethnopharmacol. 2013;145(2):465–75. 10.1016/j.jep.2012.11.010 23183090

[8] Yue R, Zhao L, Hu Y, Jiang P, Wang S, Xiang L, et al Metabolomic study of collagen-induced arthritis in rats and the interventional effects of huang-lian-jie-du-tang, a traditional chinese medicine. Evid Based Complement Alternat Med. 2013;2013:439690 10.1155/2013/439690 23533484 PMC3606714

[9] Wei Y, Shan L, Qiao L, Liu R, Hu Z, Zhang W. Protective Effects of Huang-Lian-Jie-Du-Tang against polymicrobial sepsis induced by cecal ligation and puncture in rats. Evid Based Complement Alternat Med. 2013;2013:909624 10.1155/2013/909624 24363773 PMC3865632

[10] Li CB, Li XX, Chen YG, Gao HQ, Bu PL, Zhang Y, et al Huang-lian-jie-du-tang protects rats from cardiac damages induced by metabolic disorder by improving inflammation-mediated insulin resistance. PloS one. 2013;8(6):e67530 10.1371/journal.pone.0067530 23840732 PMC3695866

[11] Lin SC, Lin CC, Lu FJ, Lin YH, Chen CH. Protective and therapeutic effects of huanglian-jie-du-tang on hepatotoxin-induced liver injuries. Am J Chin Med. 1996;24(3–4):219–29. 8982434 10.1142/S0192415X96000281

[12] Zhang XJ, Deng YX, Shi QZ, He MY, Chen B, Qiu XM. Hypolipidemic effect of the Chinese polyherbal Huanglian Jiedu decoction in type 2 diabetic rats and its possible mechanism. Phytomedicine. 2014;21(5):615–23. 10.1016/j.phymed.2013.11.004 24368167

[13] Yi Q, He XE, Luo KF, Zhang GS, Liu YH, Xue Q, et al Protection of long-term treatment with huang-lian-jie-du-tang on vascular endothelium in rats with type 2 diabetes mellitus. Curr Ther Res Clin Exp. 2012;73(6):174–85. 10.1016/j.curtheres.2012.09.002 24653519 PMC3955108

[14] Jin D, Lu FE, Chen G, Sun H, Lu XH. Effects of Huanglian Jiedu Decoction on phosphatidylinositol-3-kinase expression in target tissues of type 2 diabetic rats. J Chin integrative Med. 2007;5(5):541–5. 10.3736/jcim20070513 17854556

[15] Durairajan SS, Huang YY, Yuen PY, Chen LL, Kwok KY, Liu LF, et al Effects of Huanglian-Jie-Du-Tang and its modified formula on the modulation of amyloid-beta precursor protein processing in Alzheimer's disease models. PloS one. 2014;9(3):e92954 10.1371/journal.pone.0092954 24671102 PMC3966845

[16] Lu J, Wang JS, Kong LY. Anti-inflammatory effects of Huang-Lian-Jie-Du decoction, its two fractions and four typical compounds. J Ethnopharmacol. 2011;134(3):911–8. 10.1016/j.jep.2011.01.049 21296144

[17] Dou SS, Liu L, Jiang P, Zhang WD, Liu RH. LC-DAD and LC-ESI-MS chromatographic fingerprinting and quantitative analysis for evaluation of the quality of Huang-Lian-Jie-Du-Tang. Chromatographia. 2009;69(7–8):659–64.

[18] Yang Y, Wang HJ, Yang J, Brantner AH, Lower-Nedza AD, Si N, et al Chemical profiling and quantification of Chinese medicinal formula Huang-Lian-Jie-Du decoction, a systematic quality control strategy using ultra high performance liquid chromatography combined with hybrid quadrupole-orbitrap and triple quadrupole mass spectrometers. J Chromatogr A. 2013;1321:88–99. 10.1016/j.chroma.2013.10.072 24231264

[19] Fang H, Wang Y, Yang T, Ga Y, Zhang Y, Liu R, et al Bioinformatics analysis for the antirheumatic effects of huang-lian-jie-du-tang from a network perspective. Evid Based Complement Alternat Med. 2013;2013:245357 10.1155/2013/245357 24348693 PMC3856148

[20] Hu Y, Hu Z, Wang S, Dong X, Xiao C, Jiang M, et al Protective effects of Huang-Lian-Jie-Du-Tang and its component group on collagen-induced arthritis in rats. J Ethnopharmacol. 2013;150(3):1137–44. 10.1016/j.jep.2013.10.038 24212076

[21] Ma ZT, Yang XW, Zhang Y, Liu JX. Pharmacochemistry and integrated pharmacokinetics of six alkaloids after oral administration of huang-lian-jie-du-tang decoction. J Asian Nat Prod Res. 2014;16(5):483–96. 10.1080/10286020.2014.913577 24797560

[22] He MY, Deng YX, Shi QZ, Zhang XJ, Lv Y. Comparative pharmacokinetic investigation on baicalin and wogonoside in type 2 diabetic and normal rats after oral administration of traditional Chinese medicine Huanglian Jiedu decoction. J Ethnopharmacol. 2014;155(1):334–42. 10.1016/j.jep.2014.05.033 24910405

[23] Zhu H, Qian Z, Li H, Guo L, Pan L, Zhang Q, et al Integrated pharmacokinetics of major bioactive components in MCAO rats after oral administration of Huang-Lian-Jie-Du-Tang. J Ethnopharmacol. 2012;141(1):158–69. 10.1016/j.jep.2012.02.014 22387241

[24] Deng Y, Liao Q, Li S, Bi K, Pan B, Xie Z. Simultaneous determination of berberine, palmatine and jatrorrhizine by liquid chromatography-tandem mass spectrometry in rat plasma and its application in a pharmacokinetic study after oral administration of coptis-evodia herb couple. J Chromatogr B. 2008;863(2):195–205. 10.1016/j.jchromb.2007.12.028 18258496

[25] Deng YX, Lu T, Xie L, Liu XD. High-performance liquid chromatographic method for the determination and pharmacokinetic study of wogonoside in rat serum after oral administration of traditional Chinese medicinal preparation Huang-Lian-Jie-Du decoction. Biomed Chromatogr. 2006;20(10):1098–102. 16583457 10.1002/bmc.649

[26] Swartz ME. UPLC (TM): An introduction and review. J Liq Chromatogr R T. 2005;28(7–8):1253–63.

[27] Cai Z, Zhang Y, Pan H, Tie X, Ren Y. Simultaneous determination of 24 sulfonamide residues in meat by ultra-performance liquid chromatography tandem mass spectrometry. J Chromatogr A. 2008;1200(2):144–55. 10.1016/j.chroma.2008.05.095 18579149

[28] Flierl MA, Rittirsch D, Gao HW, Hoesel LM, Nadeau BA, Day DE, et al Adverse functions of IL-17A in experimental sepsis. Faseb J. 2008;22(7):2198–205. 10.1096/fj.07-105221 18299333

[29] Wen T, Li Y, Wu M, Sun X, Bao X, Lin Y, et al Therapeutic effects of a novel tylophorine analog, NK-007, on collagen-induced arthritis through suppressing tumor necrosis factor alpha production and Th17 cell differentiation. Arthritis Rheum. 2012;64(9):2896–906. 10.1002/art.34528 22576707

[30] Wu YH, Chuang SY, Hong WC, Lai YJ, Chang YL, Pang JH. In vivo and in vitro inhibitory effects of a traditional Chinese formulation on LPS-stimulated leukocyte-endothelial cell adhesion and VCAM-1 gene expression. J Ethnopharmacol. 2012;140(1):55–63. 10.1016/j.jep.2011.12.002 22226975

[31] Zeng H, Liu X, Dou S, Xu W, Li N, Zhang W, et al Huang-Lian-Jie-Du-Tang exerts anti-inflammatory effects in rats through inhibition of nitric oxide production and eicosanoid biosynthesis via the lipoxygenase pathway. J Pharm Pharmacol. 2009;61(12):1699–707. 10.1211/jpp/61.12.0016 19958594

[32] Miura N, Fukutake M, Yamamoto M, Ohtake N, Iizuka S, Imamura S, et al An herbal medicine orengedokuto prevents indomethacin-induced enteropathy. Biol Pharm Bull. 2007;30(3):495–501. 17329845 10.1248/bpb.30.495

[33] Dai Y, Miki K, Fukuoka T, Tokunaga A, Tachibana T, Kondo E, et al Suppression of neuropeptides' mRNA expression by herbal medicines in a rat model of peripheral inflammation. Life Sci. 2000;66(1):19–29. 10658920 10.1016/s0024-3205(99)00557-3

[34] Zeng H, Dou S, Zhao J, Fan S, Yuan X, Zhu S, et al The inhibitory activities of the components of Huang-Lian-Jie-Du-Tang (HLJDT) on eicosanoid generation via lipoxygenase pathway. J Ethnopharmacol. 2011;135(2):561–8. 10.1016/j.jep.2011.03.055 21466840

[35] Zhu HX, Pan LM, Zhang QC, Tang YP, Guo LW. Study on PK/PD model for traditional Chinese medicine biopharmaceutics based on principle of "correspondence of prescriptions and syndromes". China J Chin materia medica. 2013;38(12):2033–8. 24066607

[36] Penney M, Agoram B. At the bench: the key role of PK-PD modelling in enabling the early discovery of biologic therapies. Br J Clin Pharmacol. 2014;77(5):740–5. 10.1111/bcp.12225 23962236 PMC4004394

[37] Zuo R, Wang HJ, Si N, Zhao HY, Yang J, Bian BL. LC-FT-ICR-MS analysis of the prototypes and metabolites in rat plasma after administration of huang-lian-jie-du decoction. Acta pharmaceutica Sinica. 2014;49(2):237–43. 24761615

[38] Zuo R, Ren W, Bian BL, Wang HJ, Wang YN, Hu H, et al Metabolic fate analysis of Huang-Lian-Jie-Du Decoction in rat urine and feces by LC-IT-MS combining with LC-FT-ICR-MS: a feasible strategy for the metabolism study of Chinese medical formula. Xenobiotica. 2015:1–17. 10.3109/00498254.2015.1048541 26084375

[39] Yang Y, Zhao HY, Song JF, Wang HJ, Yang J, Si N, et al Analysis of the chemical components and neuroprotective effects of huang-lian-jie-du decoction. Lishizhen Med and Materia Medica Res. 2013;(07):1599–603.

[40] Chung HJ, Choi YH, Choi HD, Jang JM, Shim HJ, Yoo M, et al Pharmacokinetics of DA-6034, an agent for inflammatory bowel disease, in rats and dogs: Contribution of intestinal first-pass effect to low bioavailability in rats. Eur J Pharm Sci. 2006;27(4):363–74. 16387482 10.1016/j.ejps.2005.11.008

[41] Kim J, Kim SH, Lee MG. Liver and gastrointestinal first-pass effects of azosemide in rats. J Pharm Pharmacol. 1997;49(9):878–83. 9306255 10.1111/j.2042-7158.1997.tb06129.x

[42] Zeng MF, Pan LM, Zhu HX, Zhang QC, Guo LW. Comparative pharmacokinetics of baicalin in plasma after oral administration of Huang-Lian-Jie-Du-Tang or pure baicalin in MCAO and sham-operated rats. Fitoterapia. 2010;81(6):490–6. 10.1016/j.fitote.2010.01.004 20093170

[43] Deng YX, Shi QZ, Chen B, Zhang XJ, Liu SZ, Qiu XM. Comparative pharmacokinetics of baicalin in normal and the type 2 diabetic rats after oral administration of the Radix scutellariae extract. Fitoterapia. 2012;83(8):1435–42. 23339256 10.1016/j.fitote.2012.08.007

[44] Chaudhry H, Zhou J, Zhong Y, Ali MM, Mcguire F, Nagarkatti PS, et al Role of cytokines as a double-edged sword in sepsis. In Vivo. 2013;27(6):669–84. 24292568 PMC4378830

[45] Zhang L, Lin G, Chang Q, Zuo Z. Role of intestinal first-pass metabolism of baicalein in its absorption process. Pharm Res. 2005;22(7):1050–8. 16028005 10.1007/s11095-005-5303-7

[46] Xing J, Chen X, Zhong D. Absorption and enterohepatic circulation of baicalin in rats. Life Sci. 2005;78(2):140–6. 16107266 10.1016/j.lfs.2005.04.072

[47] Akao T, Kawabata K, Yanagisawa E, Ishihara K, Mizuhara Y, Wakui Y, et al Baicalin, the predominant flavone glucuronide of scutellariae radix, is absorbed from the rat gastrointestinal tract as the aglycone and restored to its original form. J Pharm Pharmacol. 2000;52(12):1563–8. 11197087 10.1211/0022357001777621

[48] Akao T, Hanada M, Sakashita Y, Sato K, Morita M, Imanaka T. Efflux of baicalin, a flavone glucuronide of Scutellariae Radix, on Caco-2 cells through multidrug resistance-associated protein 2. J Pharm Pharmacol. 2007;59(1):87–93. 17227625 10.1211/jpp.59.1.0012

[49] Tang Y, Zhu H, Zhang Y, Huang C. Determination of human plasma protein binding of baicalin by ultrafiltration and high-performance liquid chromatography. Biomed Chromatogr. 2006;20(10):1116–9. 16708379 10.1002/bmc.655

[50] Lu T, Song J, Huang F, Deng Y, Xie L, Wang G, et al Comparative pharmacokinetics of baicalin after oral administration of pure baicalin, Radix scutellariae extract and Huang-Lian-Jie-Du-Tang to rats. J Ethnopharmacol. 2007;110(3):412–8. 17110066 10.1016/j.jep.2006.09.036

[51] Zhu H, Qian Z, He F, Liu M, Pan L, Zhang Q, et al Novel pharmacokinetic studies of the Chinese formula Huang-Lian-Jie-Du-Tang in MCAO rats. Phytomedicine. 2013;20(10):767–74. 10.1016/j.phymed.2012.11.012 23628154

[52] Tsai TH, Liu SC, Tsai PL, Ho LK, Shum AY, Chen CF. The effects of the cyclosporin A, a P-glycoprotein inhibitor, on the pharmacokinetics of baicalein in the rat: a microdialysis study. Br J Pharmacol. 2002;137(8):1314–20. 12466241 10.1038/sj.bjp.0704959 PMC1573598

[53] Liu L, Deng YX, Liang Y, Pang XY, Liu XD, Liu YW, et al Increased oral AUC of baicalin in streptozotocin-induced diabetic rats due to the increased activity of intestinal beta-glucuronidase. Planta Med. 2010;76(1):70–5. 10.1055/s-0029-1185946 19639536

[54] Nishigaki J, Suzuki Y, Shigematsu A. A novel method for measuring the hepatic first-pass effect and metabolic rate of L-3,4-dihydroxyphenylalanine (DOPA), diazepam and inulin in rat liver. Biol Pharm Bull. 1998;21(7):735–40. 9703259 10.1248/bpb.21.735

[55] Ohura K, Soejima T, Nogata R, Adachi Y, Ninomiya S, Imai T. Effect of intestinal first-pass hydrolysis on the oral bioavailability of an ester prodrug of fexofenadine. J Pharm Sci. 2012;101(9):3264–74. 10.1002/jps.23182 22628163

[56] Yu KH, Lee YR, Ahn SH, Kim DD, Shim CK, Chung SJ. Contribution of a significant first-pass effect of dimethyl-4,4'-dimethoxy-5,6,5',6'-dimethylene dioxybiphenyl-2,2'-dicarboxylate in the liver to its poor bioavailability in rats. J Pharm Pharmacol. 2009;61(9):1197–203. 10.1211/jpp/61.09.0009 19703369

[57] Kang HE, Cho YK, Jung HY, Choi KY, Sohn SI, Baek SR, et al Pharmacokinetics and first-pass effects of liquiritigenin in rats: low bioavailability is primarily due to extensive gastrointestinal first-pass effect. Xenobiotica. 2009;39(6):465–75. 10.1080/00498250902890151 19480552

[58] Bae SK, Yang KH, Aryal DK, Kim YG, Lee MG. Pharmacokinetics of amitriptyline and one of its metabolites, nortriptyline, in rats: little contribution of considerable hepatic first-pass effect to low bioavailability of amitriptyline due to great intestinal first-pass effect. J Pharm Sci. 2009;98(4):1587–601. 10.1002/jps.21511 18780336

[59] Jiang HQ. Clinical observation of huang-lian-jie-du decoction to treat systemic inflammatory response syndrome. Guangming J Chin Med. 2009;24(3):480–1.

[60] Lan MH. Clinical observation of huang-lian-jie-du decoction to treat 46 cases of acute gouty arthritis. J Henan college of Tradit Chin Med. 2001;16(4):58–9.

[61] Liu GJ. Differential Treatment of 32 cases of ulcerative colitis using Traditional Chinese Medicine. Jiangxi J Tradit Chin Med. 2005;36(12):19.

[62] Xiao YL, Lu FE, Xu LJ, Leng SH, Wang KF. Protective effects of Huanglian Jiedu decoction on vascular endothelial function in type 2 diabetic rats. China J Chin materia medica. 2005;30(22):1767–70. 16468376

[63] Ma S-T, Feng C-T, Dai G-L, Song Y, Zhou G-L, Zhang X-L, et al In silico target fishing for the potential bioactive components contained in Huanglian Jiedu Tang (HLJDD) and elucidating molecular mechanisms for the treatment of sepsis. Chin J Nat Med. 2015;13(1):30–40. 10.1016/S1875-5364(15)60004-8 25660286

[64] Sharma R, Tepas JJ, Hudak ML, Mollitt DL, Wludyka PS, Teng RJ, et al Neonatal gut barrier and multiple organ failure: role of endotoxin and proinflammatory cytokines in sepsis and necrotizing enterocolitis. J Pediatr Surg. 2007;42(3):454–61. 17336180 10.1016/j.jpedsurg.2006.10.038

[65] Wang PR, Wang JS, Yang MH, Kong LY. Neuroprotective effects of Huang-Lian-Jie-Du-Decoction on ischemic stroke rats revealed by (1)HNMR metabolomics approach. J Pharm Biomed Anal. 2014;88:106–16. 10.1016/j.jpba.2013.08.025 24051274

[66] Wang LM, Yamamoto T, Wang XX, Yang L, Koike Y, Shiba K, et al Effects of Oren-gedoku-to and Unsei-in, Chinese traditional medicines, on interleukin-8 and superoxide dismutase in rats. J Pharm Pharmacol. 1997;49(1):102–4. 9120759 10.1111/j.2042-7158.1997.tb06760.x

[67] Li G, Zhao H, Yang J. Research progress on current pharmacokinetic evaluation of Chinese herbal medicines. China J Chin materia medica. 2011;36(5):644–9. 21657088

