# Supplementary material for: Pharmacokinetic-Pharmacodynamic Analysis on Inflammation Rat Model after Oral Administration of Huang Lian Jie Du Decoction
Source: PLoS One. 2016 Jun 9;11(6):e0156256. doi: 10.1371/journal.pone.0156256 (PMC4900566; doi:10.1371/journal.pone.0156256)
Supplement: S1 Fig — Samples from left to right are rat plasma after oral administration of HLJDD, extract of HLJDD and blank plasma of rats. (PDF) [file pone.0156256.s001.pdf]

I_1 (gardenoside)

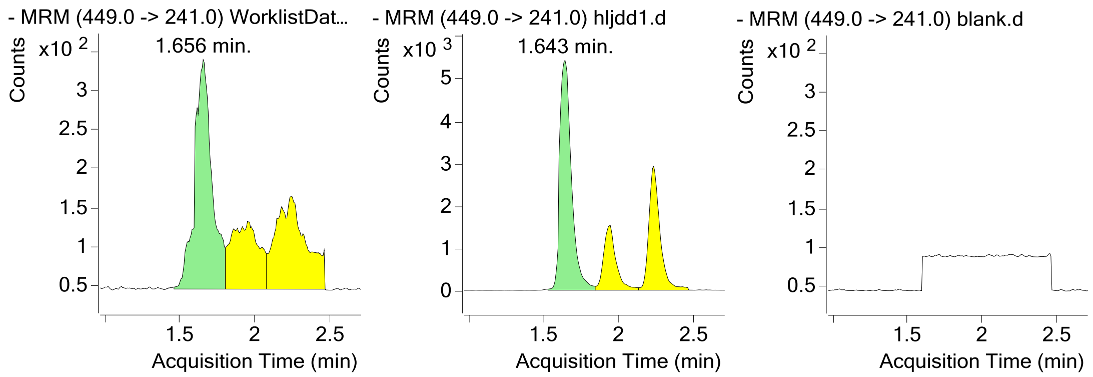

A_1 (phellodendrine-O-glucuronide)

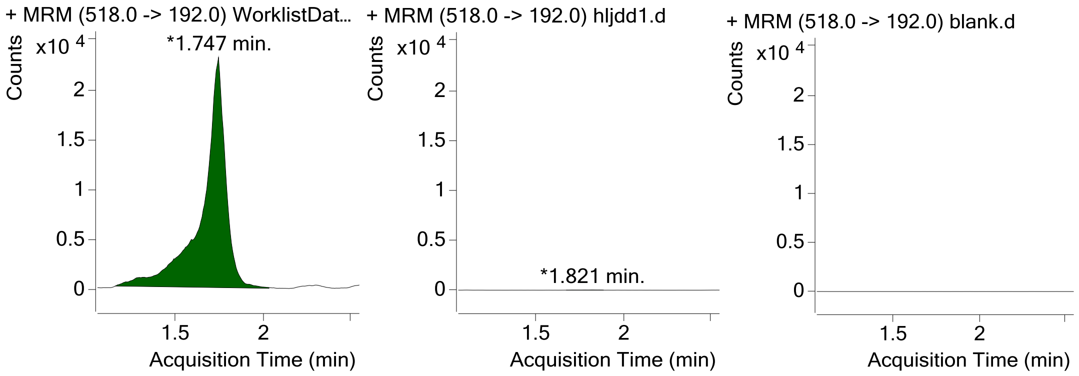

I_2 (scandoside methyl ester)

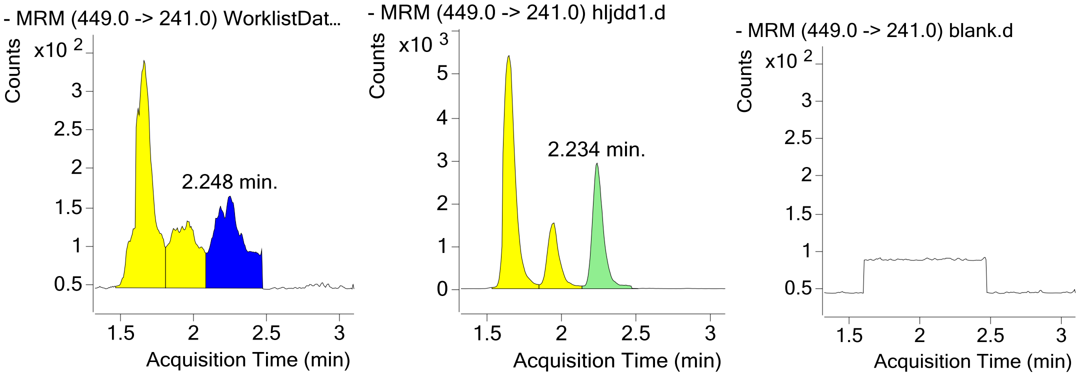

I_3 (genipin-1-gentiobioside)

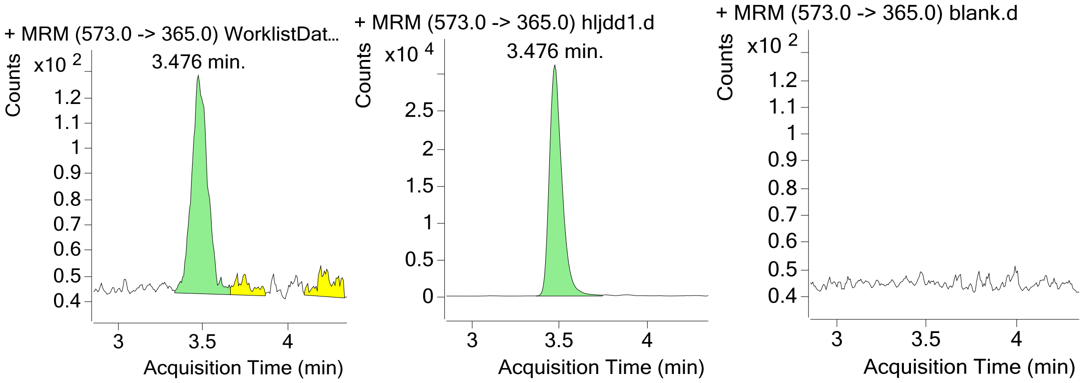

A$_2$ (phellodendrine)

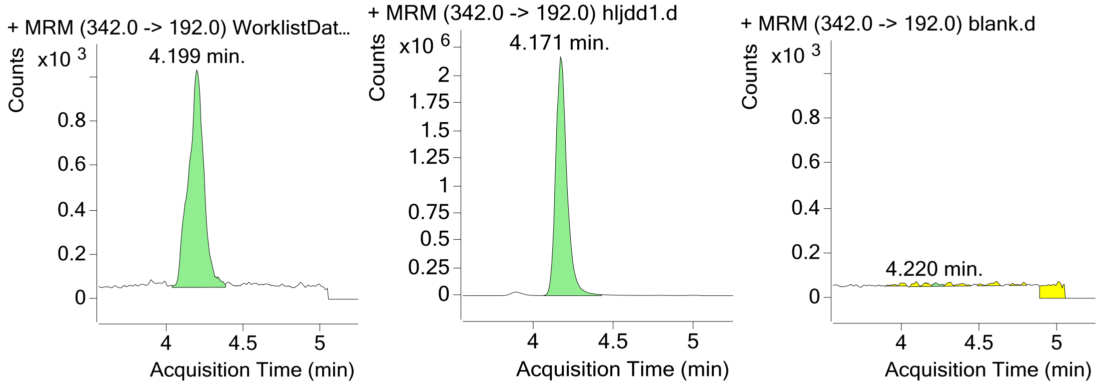

I$_4$ (geniposide)

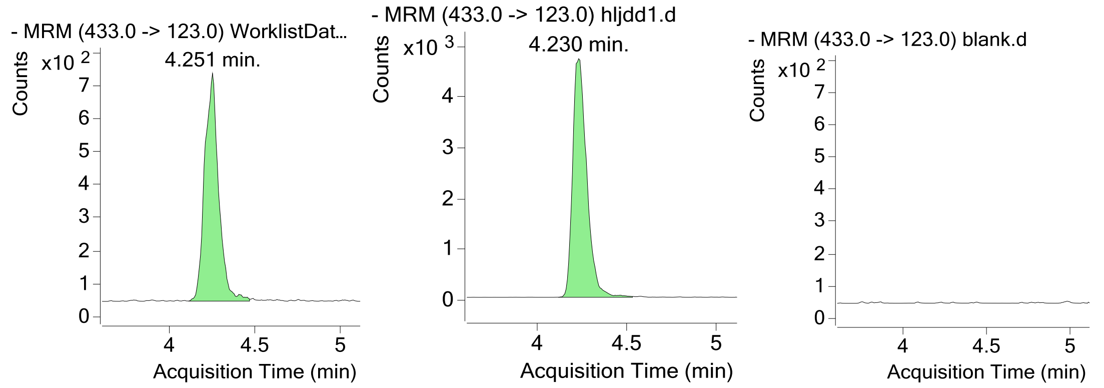

A$_3$/A$_4$ (etradehydroscoulerine-O-glucuronide AND groenlandicine-O-glucuronide)

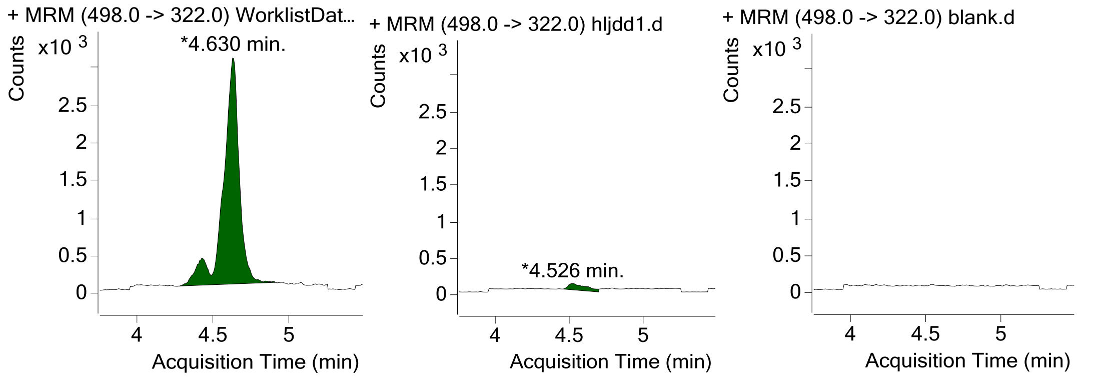

A$_5$ (demethylenecoptisine-di-O-glucuronide)

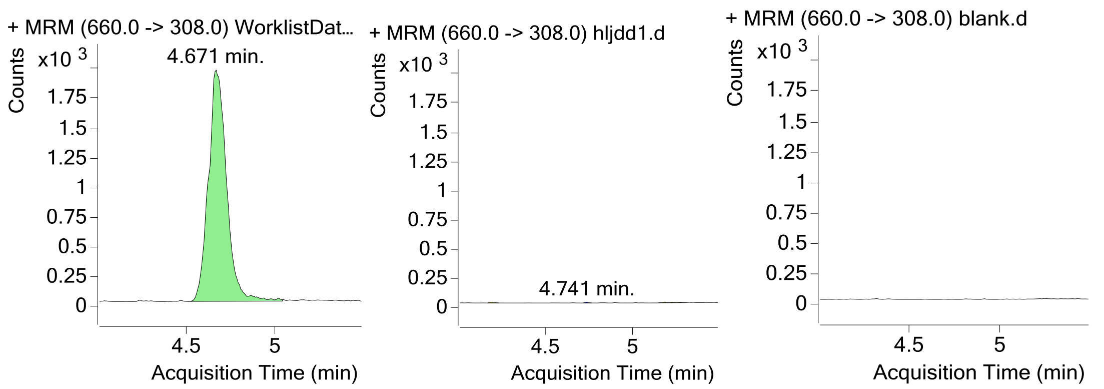

A_6 (magnolflorine)

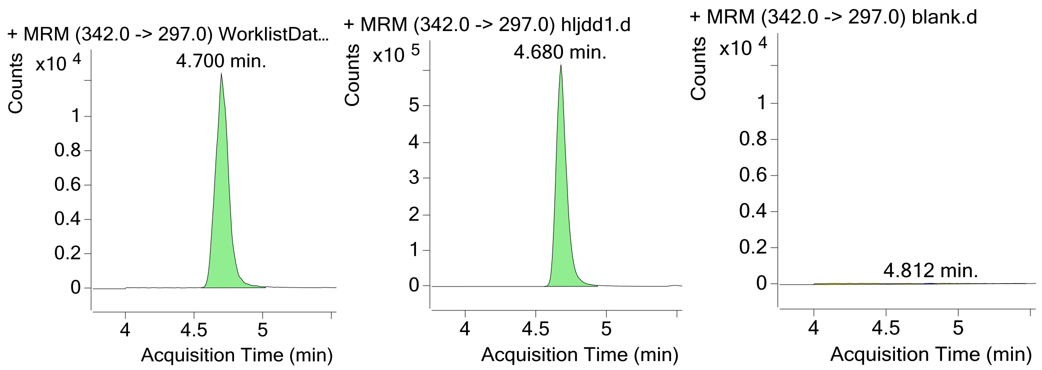

A_7/A_8 (demethyleneepiberberine-O-glucuronide)

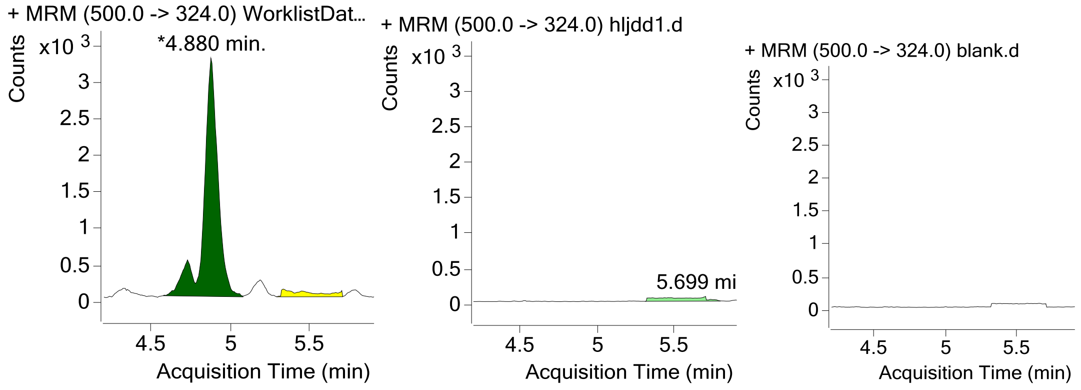

A_9 (demethyleneberberine-O-glucuronide)

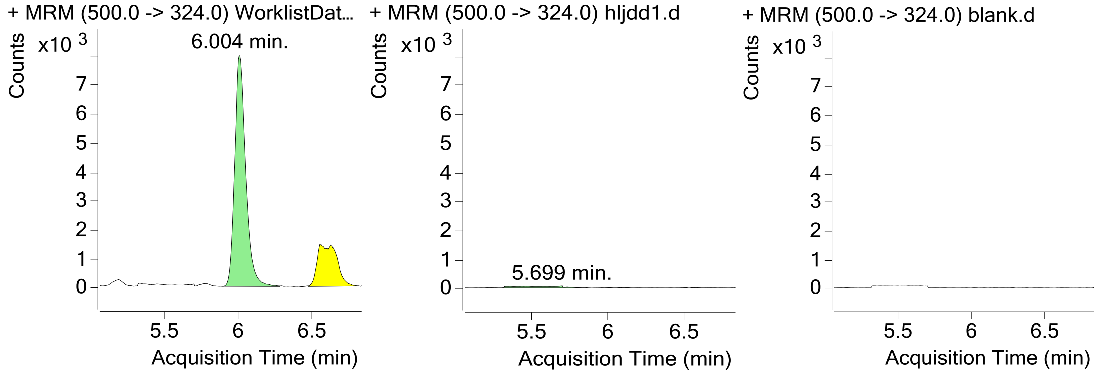

A_{10} (jatrorrhizine-O-glucuronide)

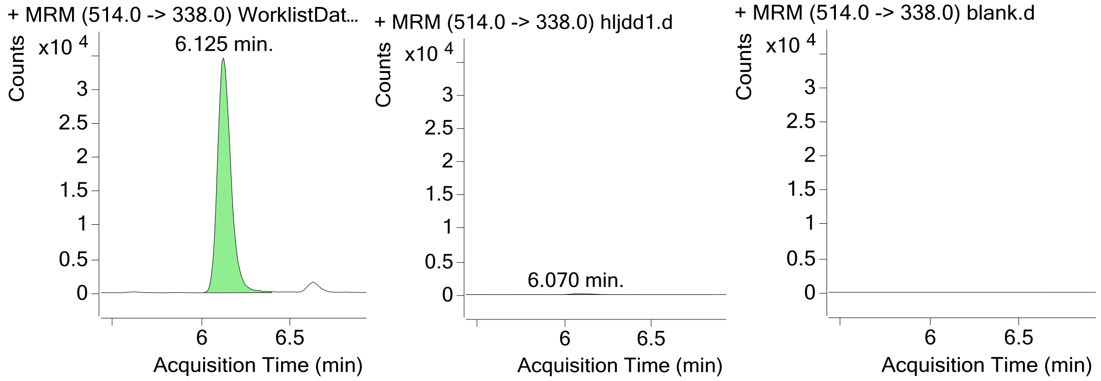

A_{11} (thalifendin-O-glucuronide)

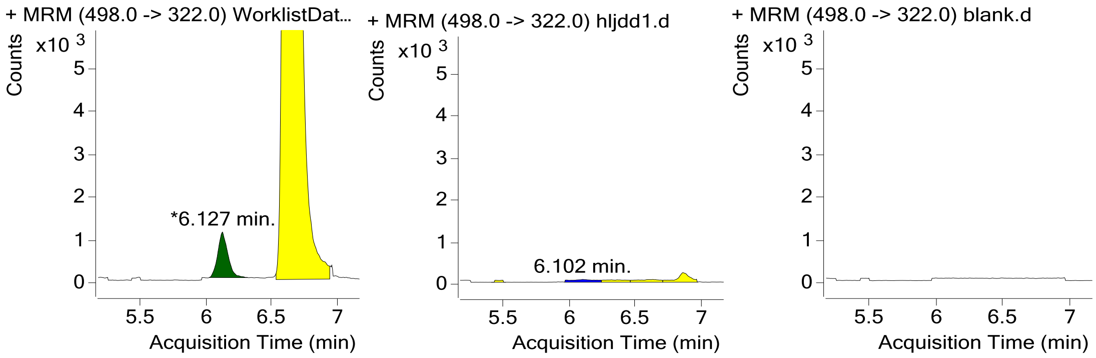

A$_{12}$ (berberrubinen-O-glucuronide)

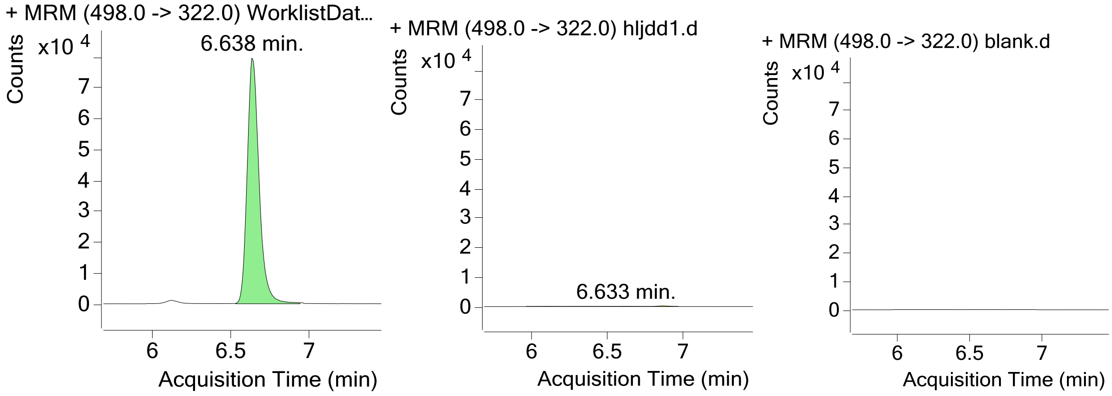

A$_{13}$ (demethylenecoptisine-O-glucuronide)

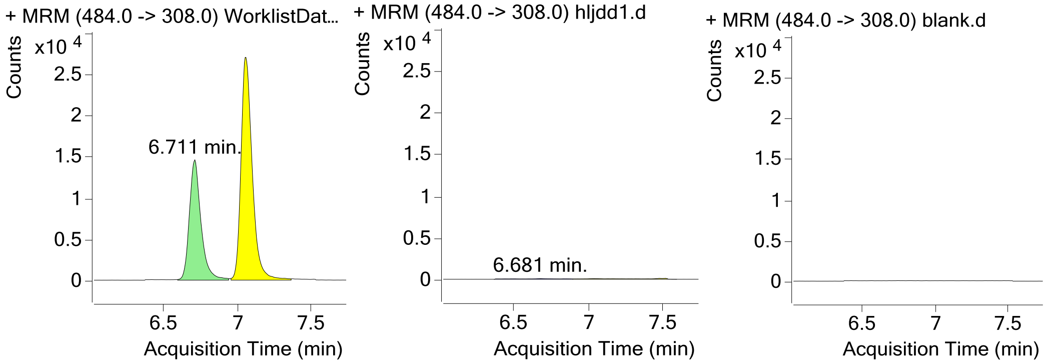

A$_{14}$ (demethylenecoptisine-O-glucuronide)

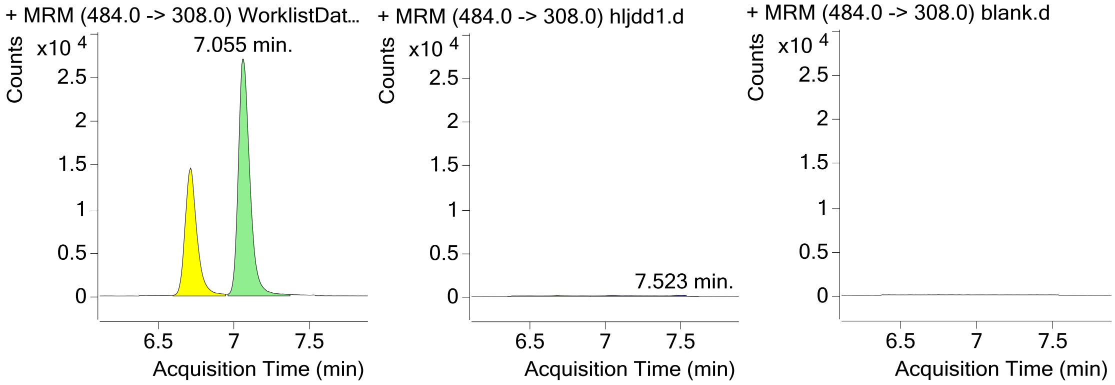

A$_{15}$ (groenlandicine)

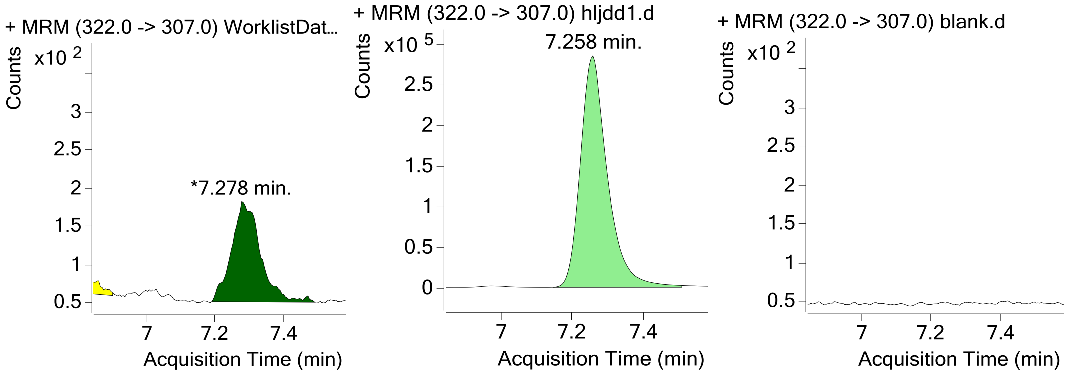

F1 (5,6,7-trihyroxy-8-methoxy flavone-7-O-glucuronide)

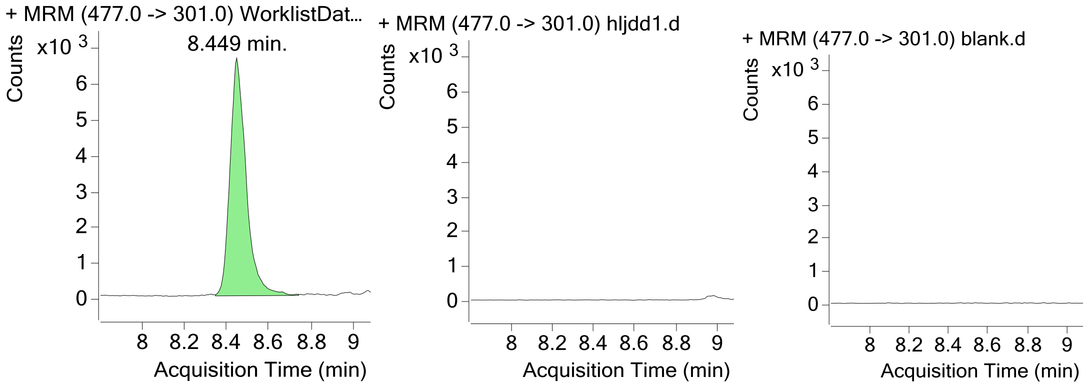

F2/F3 (baicalein-5,7-di-O-glucuronide AND baicalein-6,7-di-O-glucuronide)

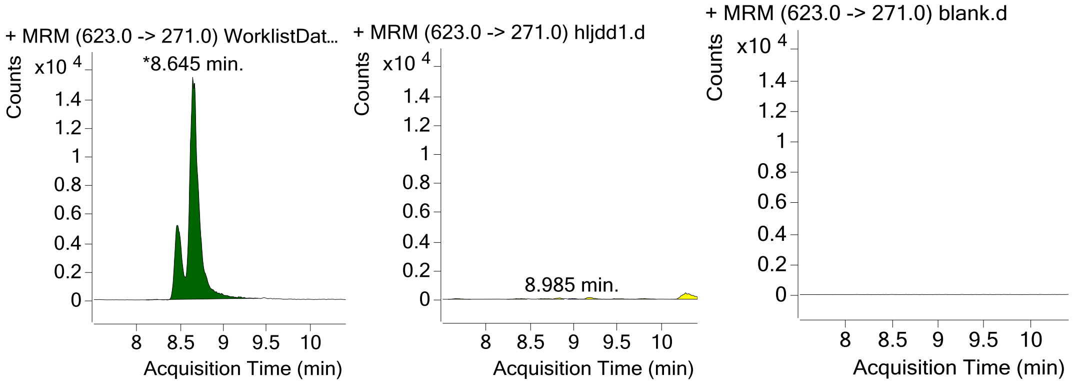

A16 (columbamine)

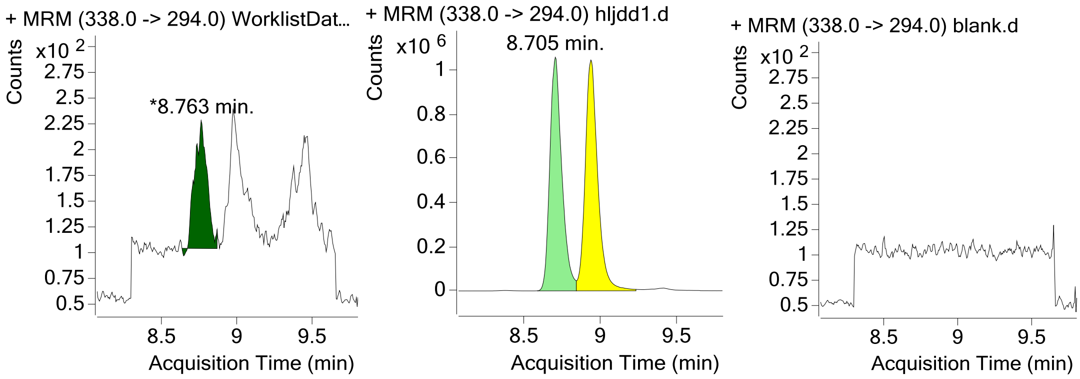

A17 (epiberberine)

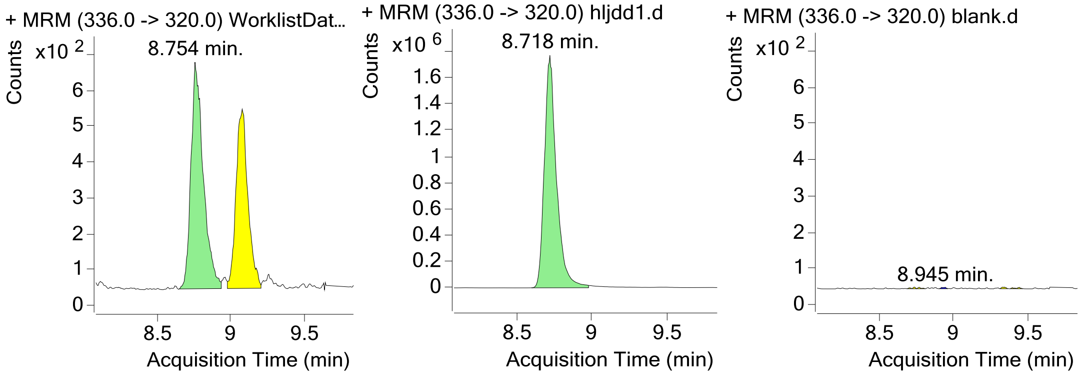

A_{18} (coptisine)

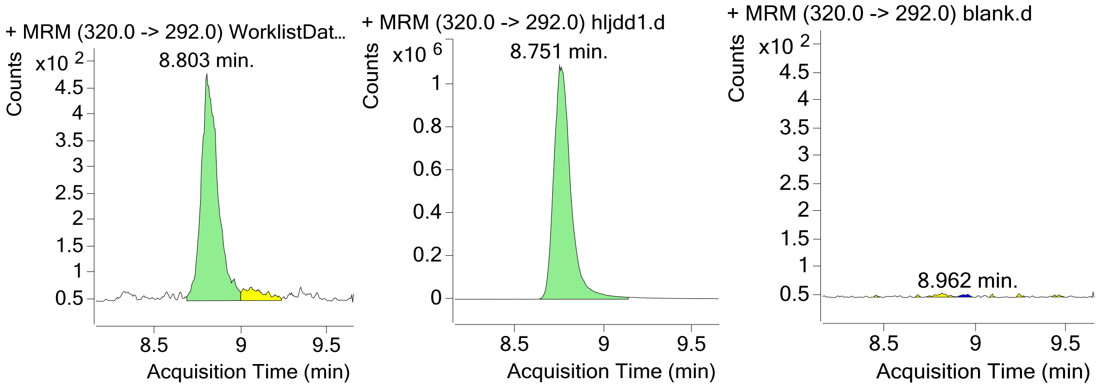

A_{19} (jatrorrhizine)

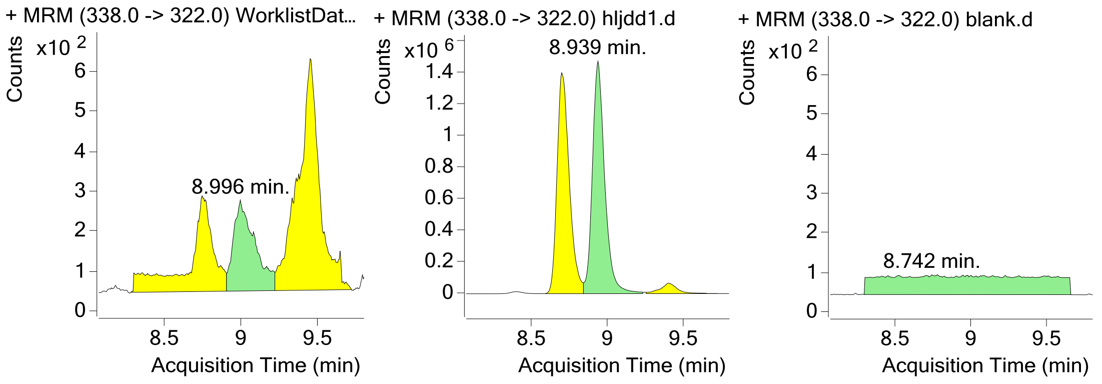

F_4 (5,8-dihyroxy-7-methoxy flavone-5,8-di-O-glucuronide)

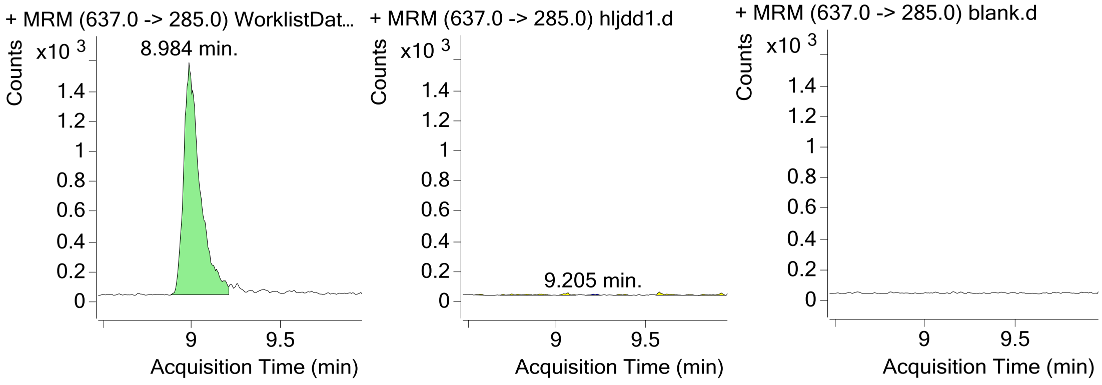

F_5 (5,6,7-trihyroxy-8-methoxy flavone-6,7-di-O-glucuronide)

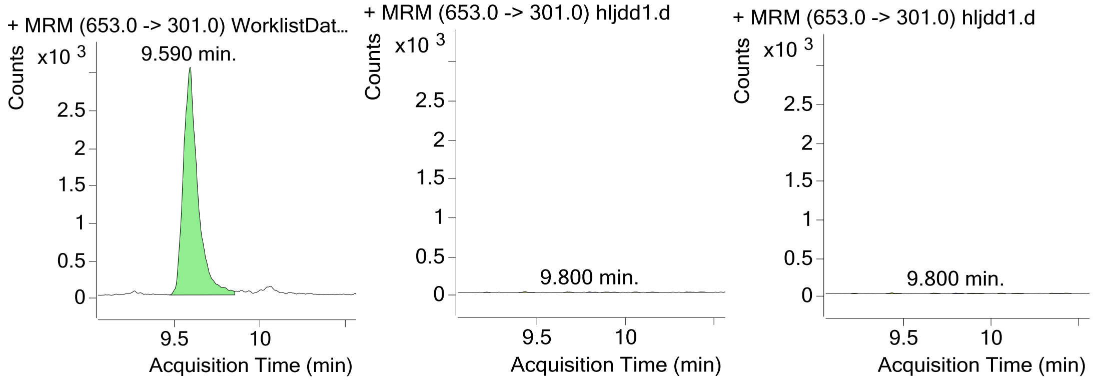

A$_{20}$ (thalifendin OR berberrubinen)

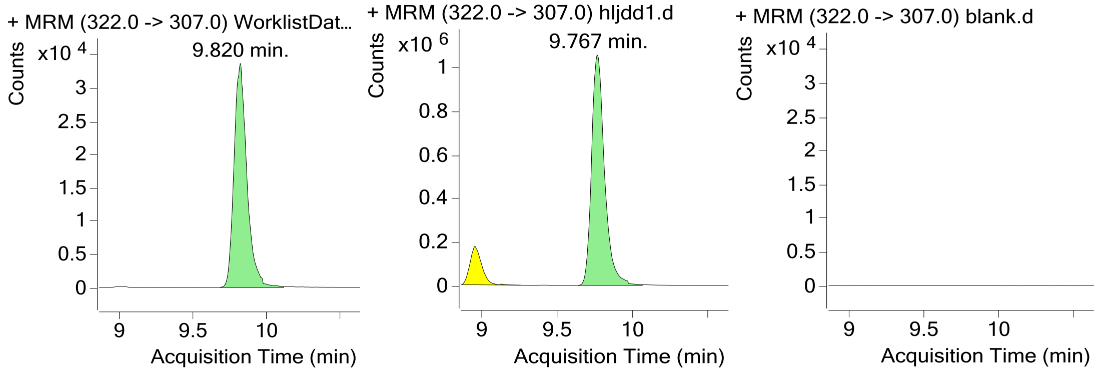

F$_{6}$ (baicalin)

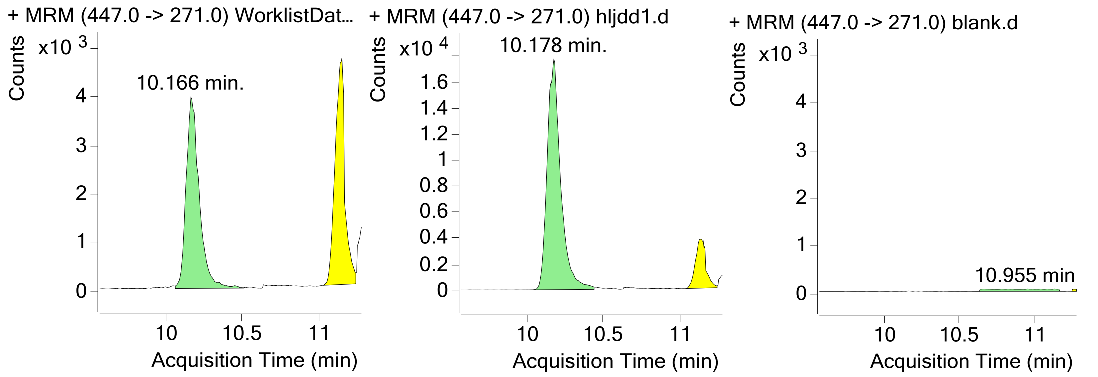

A$_{21}$ (palmatine)

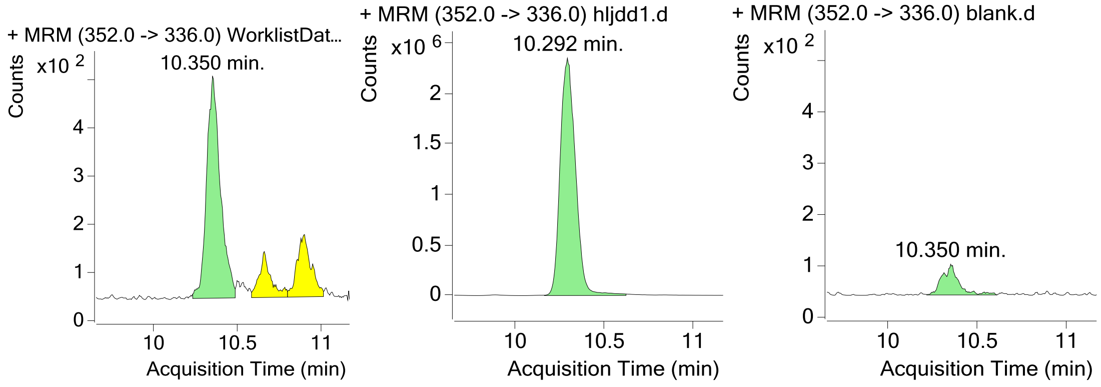

A$_{22}$ (berberine)

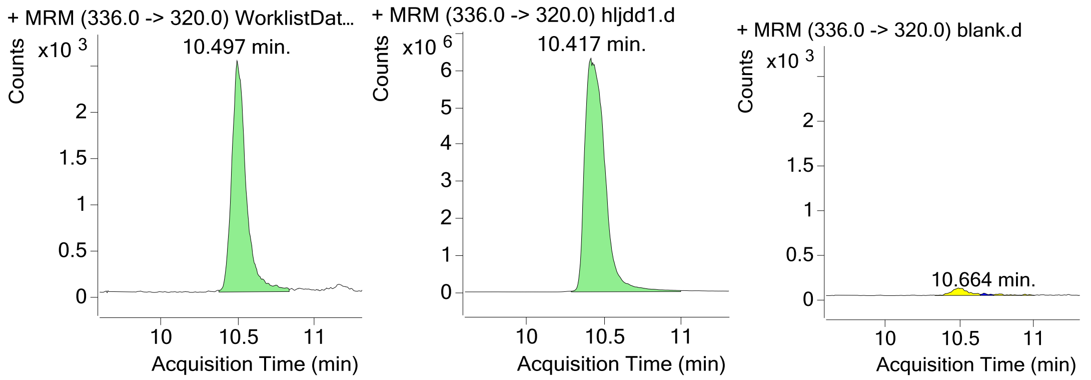

F_7 (5,8-dihyroxy-7-methoxy flavone-8-O-glucuronide)

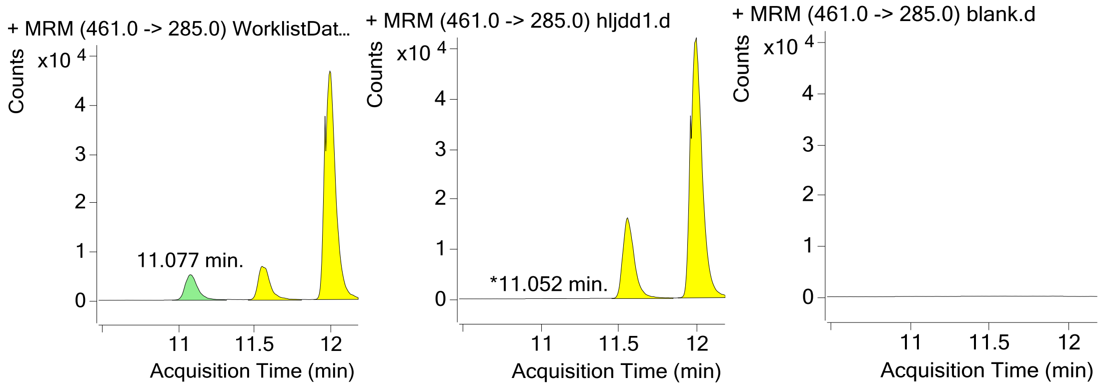

F_8/F_9 (wogonin-7-O-glucoside AND norwogonin-7-O-glucuronide)

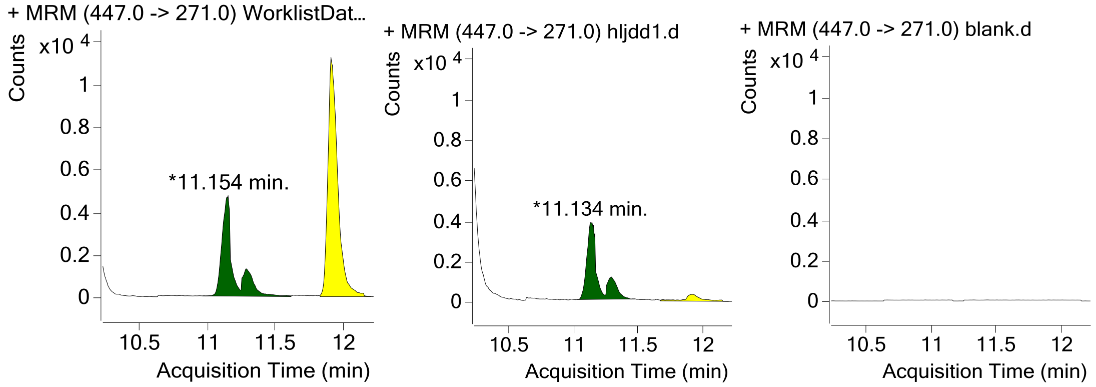

F_{10} (oroxylin A 7-O-glucuronide)

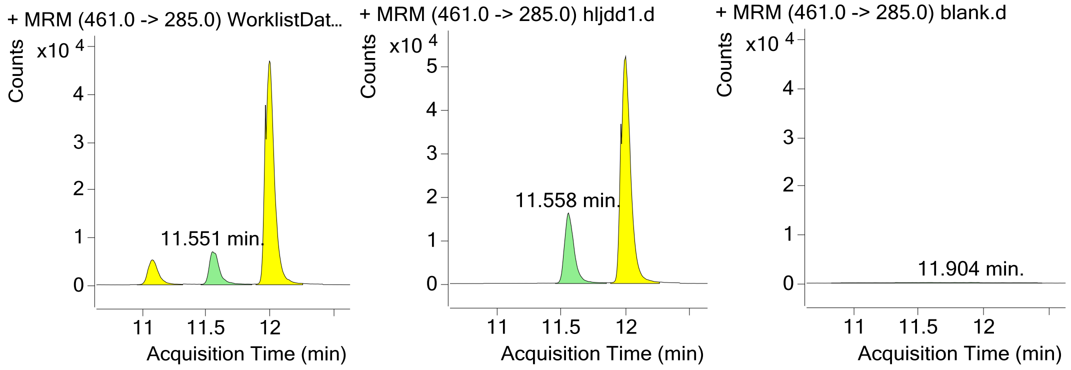

F_{11} (trihyroxy-methoxy-flavone-O-glucuronide)

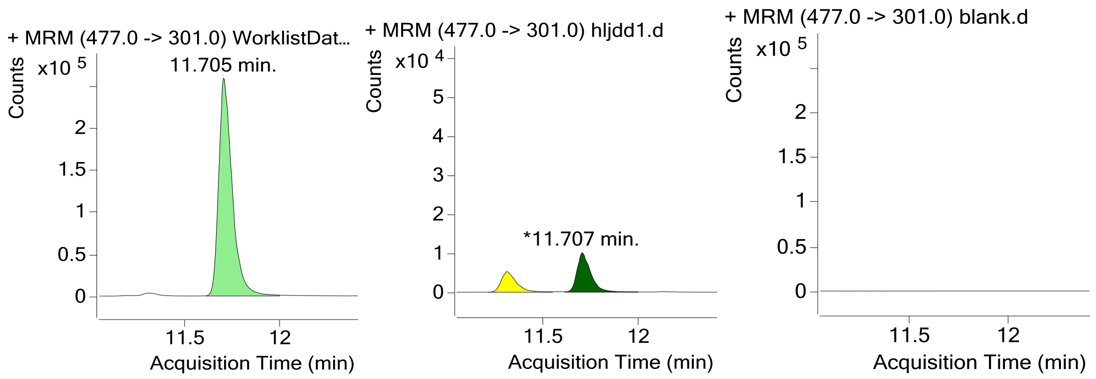

F12 (baicalein-6-O-glucuronide)

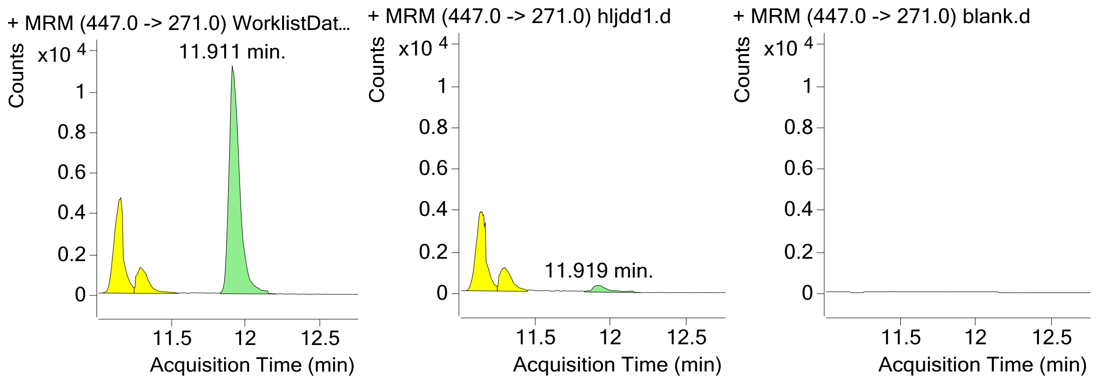

F13 (wogonoside)

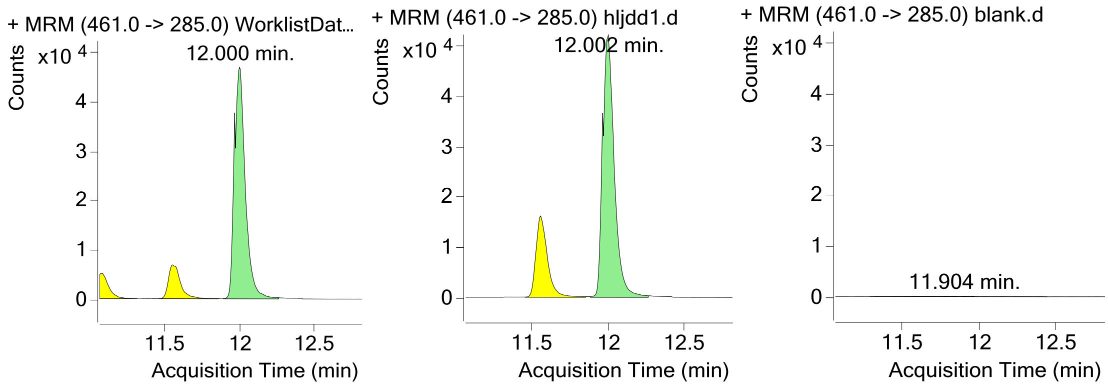

F14 (wogonin)

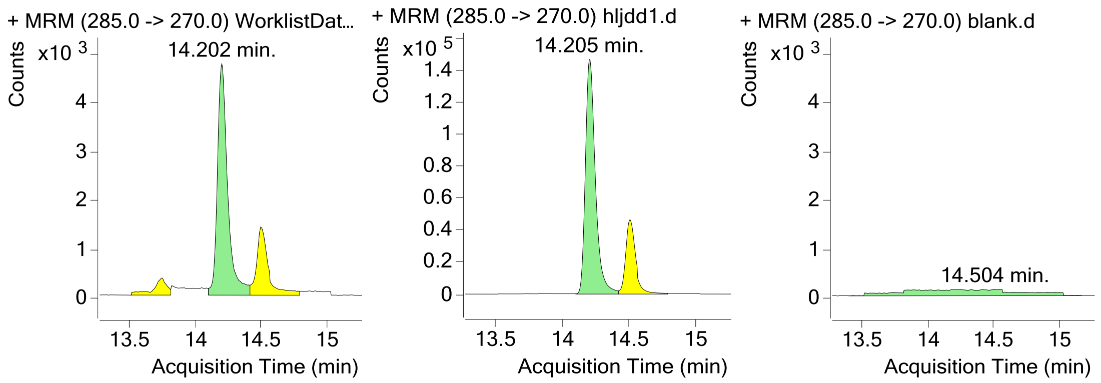

F15 (oroxylin A)

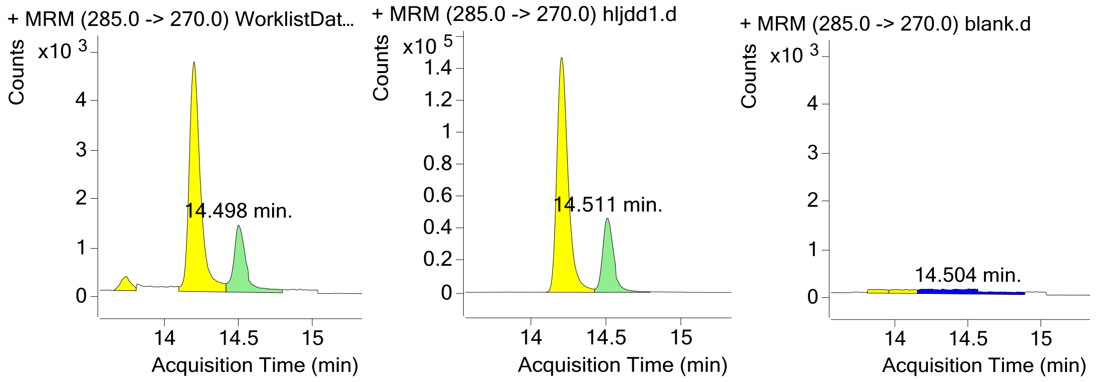

S1 Fig. The extracted chromatograms of 41 constituents in samples. Samples from left to right are rat plasma after oral administration of HLJDD, extract of HLJDD and blank plasma of rats.
