# Supplementary material for: Pharmacokinetic-Pharmacodynamic Analysis on Inflammation Rat Model after Oral Administration of Huang Lian Jie Du Decoction
Source: PLoS One. 2016 Jun 9;11(6):e0156256. doi: 10.1371/journal.pone.0156256 (PMC4900566; doi:10.1371/journal.pone.0156256)
Supplement: S2 Fig — (PDF) [file pone.0156256.s002.pdf]

I

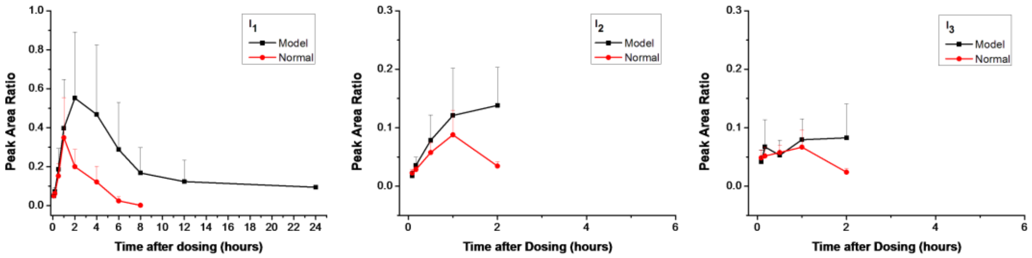

I1
Model
Normal
Peak Area Ratio
Time after dosing (hours)
I2
Model
Normal
Peak Area Ratio
Time after Dosing (hours)
I3
Model
Normal
Peak Area Ratio
Time after Dosing (hours)

A

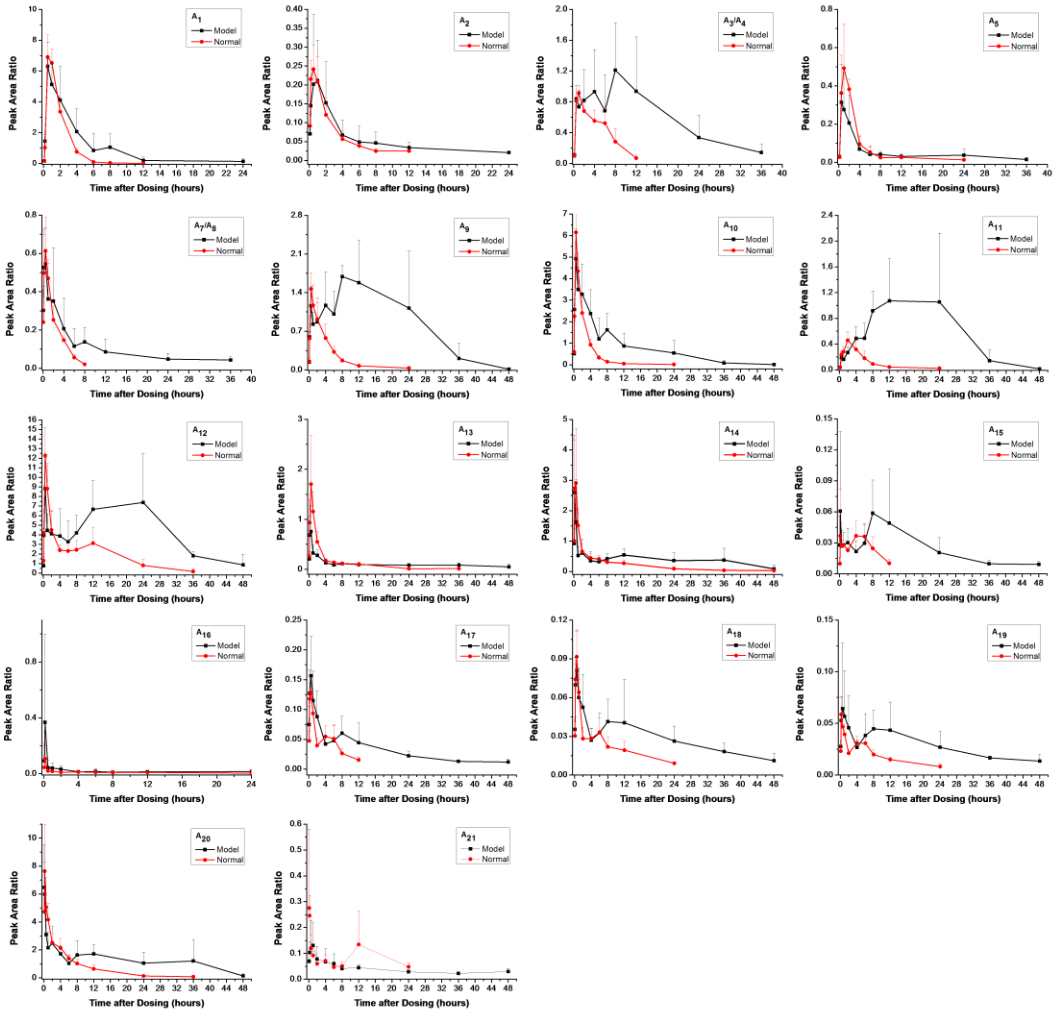

A1
A2
A3/A4
A5
A7/A8
A9
A10
A11
A12
A13
A14
A15
A16
A17
A18
A19
A20
A21
Model
Normal
Peak Area Ratio
Time after Dosing (hours)

F

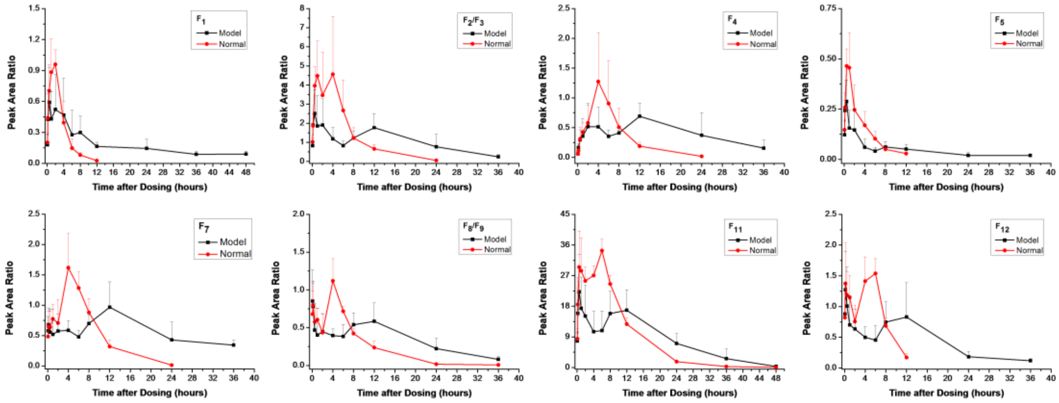

S2 Fig. The peak area ratio-time curves of the other 33 ingredients in normal and inflammation rats. I, iridoids; A, alkaloids; F, flavonoids.
