# Supplementary material for: Pharmacokinetic-Pharmacodynamic Analysis on Inflammation Rat Model after Oral Administration of Huang Lian Jie Du Decoction
Source: PLoS One. 2016 Jun 9;11(6):e0156256. doi: 10.1371/journal.pone.0156256 (PMC4900566; doi:10.1371/journal.pone.0156256)
Supplement: S1 Table — (DOCX) [file pone.0156256.s003.docx]

**S1 Table. The selected detecting ions, collision energy (CE) and detecting conditions of the analytes**

| **No.** | **Classes** | **P/ M/IS** | **Test Code** | **Chemical Compounds** | **Quant/Qual**  **I**[**on**](javascript:void(0);)**Pair** | **Quant**  **/Qual**  **CE (V)** | **RT**  **(min)** | **Scan Width**  **(min)** | **Ion Mode** |
| --- | --- | --- | --- | --- | --- | --- | --- | --- | --- |
| **1** | I_1_ | P | 449-1 | gardenoside | 449→241/449→403 | 8/20 | 1.7 | 1.5 | - |
| **2** | A_1_ | M | 518 | phellodendrine-O-glucuronide | 518→342/518→192 | 28/35 | 1.7 | 1.5 | + |
| **3** | I_2_ | P | 449-2 | scandoside methyl ester | 449→241/449→403 | 8/20 | 2.2 | 1.5 | - |
| **4** | I_3_ | P | 573 | genipin-1-gentiobioside | 573→365/573→347 | 40/40 | 3.5 | 1.5 | + |
|  | I | IS | 419 | swertiamarin | 419→179 | 8 | 3.5 | 1.5 | - |
| **5** | A_2_ | P | 342-1 | phellodendrine | 342→192/342→177 | 24/50 | 4.2 | 1.5 | + |
| **6** | I_4_ | P | 433 | geniposide | 433→123/433→225 | 8/20 | 4.3 | 1.5 | - |
| **7/8** | A_3_/A_4_ | M/M | 498-1,2 | etradehydroscoulerine-O-glucuronide and groenlandicine-O-glucuronide | 498→322 | 32 | 4.4/4.6 | 1.5 | + |
| **9** | A_5_ | M | 660 | demethylenecoptisine-di-O-glucuronide | 660→308 | 44 | 4.7 | 1.5 | + |
| **10** | A_6_ | P | 342-2 | magnolflorine | 342→297/342→265 | 20/20 | 4.7 | 1.5 | + |
| **11/12** | A_7_ /A_8_ | M/M | 500-1,2 | demethyleneepiberberine-O-glucuronide | 500→324 | 32 | 4.7/4.9 | 1.5 | + |
| **13** | A_9_ | M | 500-3 | demethyleneberberine-O-glucuronide | 500→324 | 32 | 6.0 | 1.5 | + |
| **14** | A_10_ | M | 514 | jatrorrhizine-O-glucuronide | 514→338 | 32 | 6.1 | 1.5 | + |
| **15** | A_11_ | M | 498-3 | thalifendin-O-glucuronide and  berberrubinen-O-glucuronide | 498→322 | 32 | 6.1 | 1.5 | + |
| **16** | A_12_ | M | 498-4 |  | 498→322 | 32 | 6.6 | 1.5 | + |
| **17** | A_13_ | M | 484-1 | demethylenecoptisine-O-glucuronide | 484→308 | 20 | 6.7 | 1.5 | + |
| **18** | A_14_ | M | 484-2 |  | 484→308 | 20 | 7.1 | 1.5 | + |
| **19** | A_15_ | P | 322-1 | groenlandicine | 322→307 | 28 | 7.3 | 1.5 | + |
| **20** | F_1_ | P | 477-1 | 5,6,7-trihyroxy-8-methoxy flavone-7-O-glucuronide | 477→301 | 24 | 8.5 | 1.5 | + |
| **21/22** | F_2_ /F_3_ | M/M | 623-1,2 | baicalein-5,7-di-O-glucuronide and baicalein-6,7-di-O-glucuronide | 623→271 | 40 | 8.5/8.6 | 3.0 | + |
| **23** | A_16_ | P | 338-1 | columbamine | 338→322/338→294 | 32/32 | 8.7 | 1.5 | + |
| **24** | A_17_ | P | 336-1 | epiberberine | 336→320/336→292 | 36/36 | 8.7 | 1.5 | + |
| **25** | A_18_ | P | 320 | coptisine | 320→292/320→277 | 36/40 | 8.8 | 1.5 | + |
| **26** | A_19_ | P | 338-2 | jatrorrhizine | 338→322/338→294 | 32/32 | 9.0 | 1.5 | + |
| **27** | F_4_ | M | 637 | 5,8-dihyroxy-7-methoxy flavone-5,8-di-O-glucuronide | 637→285 | 40 | 9.0 | 1.5 | + |
| **28** | F_5_ | M | 653 | 5,6,7-trihyroxy-8-methoxy flavone-6,7-di-O-glucuronide | 653→301 | 44 | 9.6 | 1.5 | + |
| **29** | A_20_ | P | 322-2 | thalifendin or berberrubinen | 322→307 | 28 | 9.8 | 1.5 | + |
| **30** | F_6_ | P | 447-1 | baicalin | 447→271/447→253 | 75/20 | 10.2 | 1.5 | + |
|  | A | IS | 368 | corynoline | 368→337 | 20 | 10.2 | 1.5 | + |
| **31** | A_21_ | P | 352 | palmatine | 352→336/352→308 | 36/38 | 10.4 | 1.5 | + |
| **32** | A_22_ | P | 336-2 | berberine | 336→320/336→292 | 36/36 | 10.5 | 1.5 | + |
| **33** | F_7_ | M | 461-1 | 5,8-dihyroxy-7-methoxy flavone-8-O-glucuronide | 461→285/461→270 | 50/20 | 11.1 | 1.5 | + |
| **34/35** | F_8_/F_9_ | P/P | 447-2,3 | wogonin-7-O-glucoside and  norwogonin-7-O-glucuronide | 447→271/447→253 | 75/20 | 11.2/11.3 | 1.5 | + |
| **36** | F_10_ | P | 461-2 | oroxylin A 7-O-glucuronide | 461→285/461→270 | 50/20 | 11.6 | 1.5 | + |
| **37** | F_11_ | M/P | 477-2 | trihyroxy-methoxy-flavone-O-glucuronide | 477→301 | 24 | 11.7 | 1.5 | + |
| **38** | F_12_ | M/P | 447-4 | baicalein-6-O-glucuronide | 447→271/447→253 | 75/20 | 11.9 | 1.5 | + |
| **39** | F_13_ | P | 461-3 | wogonoside | 461→285/461→270 | 50/20 | 12.0 | 1.5 | + |
|  | F | IS | 677 | icariin | 677→531/677→369 | 25/28 | 12.1 | 1.5 | + |
| **40** | F_14_ | P | 285-1 | wogonin | 285→270/285→168 | 40/44 | 14.2 | 1.5 | + |
| **41** | F_15_ | P | 285-2 | oroxylin A | 285→270/285→168 | 40/44 | 14.5 | 1.5 | + |

I, iridoids; A, alkaloids; F, flavonoids; P, prototype; M, metabolite; IS, internal standard; Quant, quantitative; Qual, qualitative
