# Supplementary material for: Pharmacokinetic-Pharmacodynamic Analysis on Inflammation Rat Model after Oral Administration of Huang Lian Jie Du Decoction
Source: PLoS One. 2016 Jun 9;11(6):e0156256. doi: 10.1371/journal.pone.0156256 (PMC4900566; doi:10.1371/journal.pone.0156256)
Supplement: S2 Table — (DOCX) [file pone.0156256.s004.docx]

**S2 Table. Calibration curves and LOQ of geniposide, magnolflorine, baicalin, berberine, oroxylin A­7­O­glucuronide, wogonoside, wogonin and oroxylin A**

| **Analyte** | **Regression Equations** | **R^2^** | **Linear Range** | **Weight** | **LOQ** |
| --- | --- | --- | --- | --- | --- |
| **Geniposide (I_4_)** | Y=15.055178*X+0.001387 | 0.9939 | 5.36-10720.0 ng/mL | 1/X^2^ | 5.36 ng/mL |
| **Magnolflorine (A_6_)** | Y=41.306282*X+0.018388 | 0.9962 | 0.11-223.20 ng/mL | 1/X^2^ | 0.11 ng/mL |
| **Baicalin (F_6_)** | Y=2.128158*X-0.015686 | 0.9802 | 20.30-40600.00 ng/mL | 1/X^2^ | 20.30 ng/mL |
| **Berberine (A_22_)** | Y=92.075885*X+0.082455 | 0.9837 | 7.50-15004.80 pg/mL | 1/X^2^ | 7.50 pg/mL |
| **Oroxylin A-7-O-glucuronide (F_10_)** | Y=64.0456*X+0.173711 | 0.9828 | 0.70-1390.00 ng/mL | 1/X^2^ | 0.70 ng/mL |
| **Wogonoside (F_13_)** | Y=55.819101*X+0.001021 | 0.9975 | 5.54-11080.00 ng/mL | 1/X^2^ | 5.54 ng/mL |
| **Wogonin (F_14_)** | Y=3230.684100*X-0.197526 | 0.9917 | 0.22-440.00 ng/mL | 1/X^2^ | 0.088 ng/mL |
| **Oroxylin A (F_15_)** | Y=886.246773*X+0.079796 | 0.9940 | 0.15-299.20 ng/mL | 1/X^2^ | 0.06 ng/mL |
