# Supplementary material for: Pharmacokinetic-Pharmacodynamic Analysis on Inflammation Rat Model after Oral Administration of Huang Lian Jie Du Decoction
Source: PLoS One. 2016 Jun 9;11(6):e0156256. doi: 10.1371/journal.pone.0156256 (PMC4900566; doi:10.1371/journal.pone.0156256)
Supplement: S3 Table — (DOCX) [file pone.0156256.s005.docx]

**S3 Table. Precision and accuracy of geniposide, magnolflorine, baicalin, berberine, oroxylin A­7­O­glucuronide, wogonoside, wogonin and oroxylin A**

| **Components** | **Spiked Concentration (ng/mL)** | **Intra-day (n=6)** | | | | **Inter-day (n=6)** | | |
| --- | --- | --- | --- | --- | --- | --- | --- | --- |
|  |  | **Measured Concentration (ng/mL, mean****±SD)** | **RSD (%)** | **RE (%)** | **Measured Concentration (mean±SD, ng/mL)** | | **RSD (%)** | **RE (%)** |
| **Geniposide** | 22.00 | 22.96±1.82 | 7.9 | 4.3 | | 22.39±1.36 | 6.1 | 1.8 |
|  | 110.00 | 118.03±4.18 | 3.5 | 7.3 | | 117.27±1.18 | 1.0 | 6.6 |
|  | 1100.00 | 1115.84±18.16 | 1.6 | 1.4 | | 1095.13±32.06 | 2.9 | -0.4 |
| **Magnolflorine** | 0.44 | 0.425±0.015 | 3.5 | -3.4 | | 0.462±0.032 | 6.9 | 5.0 |
|  | 2.20 | 2.166±0.034 | 1.6 | -1.5 | | 2.095±0.053 | 2.5 | -4.8 |
|  | 22.00 | 24.526±0.697 | 2.8 | 11.5 | | 24.425±1.109 | 4.5 | 11.0 |
| **Baicalin** | 80.00 | 65.67±0.30 | 0.5 | -17.9 | | 68.97±3.14 | 4.6 | -13.8 |
|  | 400.00 | 352.37±7.77 | 2.2 | -11.9 | | 349.49±11.71 | 3.4 | -12.6 |
|  | 4000.00 | 4620.80±86.7 | 1.9 | 15.5 | | 4591.69±31.04 | 0.7 | 14.8 |
| **Berberine** | 0.03 | 0.031±0.003 | 9.7 | 3.3 | | 0.032±0.002 | 6.3 | 6.7 |
|  | 0.15 | 0.161±0.007 | 4.3 | 7.3 | | 0.164±0.009 | 5.5 | 9.3 |
|  | 1.50 | 1.587±0.029 | 1.8 | 5.8 | | 1.589±0.052 | 3.3 | 5.9 |
| **Oroxylin A-7-O-glucuronide** | 2.80 | 2.87±0.09 | 3.1 | 2.5 | | 2.73±0.31 | 11.4 | -2.5 |
|  | 14.00 | 12.79±1.29 | 10.1 | -8.6 | | 12.67±1.06 | 8.4 | -9.5 |
|  | 140.00 | 145.82±2.11 | 1.4 | 4.2 | | 149.75±5.54 | 3.7 | 7.0 |
| **Wogonoside** | 22.00 | 24.88±0.36 | 1.4 | 13.1 | | 23.75±0.99 | 4.2 | 8.0 |
|  | 110.00 | 96.29±2.03 | 2.1 | -12.5 | | 96.14±2.45 | 2.5 | -12.6 |
|  | 1100.00 | 1151.34±17.08 | 1.5 | 4.7 | | 1166.43±26.74 | 2.3 | 6.0 |
| **Wogonin** | 0.88 | 0.93±0.02 | 2.2 | 5.7 | | 0.95±0.05 | 5.3 | 8.0 |
|  | 4.40 | 4.90±0.12 | 2.4 | 11.4 | | 4.72±0.34 | 7.2 | 7.3 |
|  | 44.00 | 48.32±0.89 | 1.8 | 9.8 | | 48.40±1.53 | 3.2 | 2.5 |
| **Oroxylin A** | 0.54 | 0.59±0.03 | 5.1 | 9.3 | | 0.53±0.04 | 7.5 | -1.9 |
|  | 2.70 | 3.01±0.15 | 5.0 | 11.5 | | 2.77±0.19 | 6.9 | 2.6 |
|  | 27.00 | 28.36±0.55 | 1.9 | 5.0 | | 28.20±1.49 | 5.3 | 4.4 |
