# Supplementary material for: Pharmacokinetic-Pharmacodynamic Analysis on Inflammation Rat Model after Oral Administration of Huang Lian Jie Du Decoction
Source: PLoS One. 2016 Jun 9;11(6):e0156256. doi: 10.1371/journal.pone.0156256 (PMC4900566; doi:10.1371/journal.pone.0156256)
Supplement: S4 Table — (DOCX) [file pone.0156256.s006.docx]

**S4 Table. Stability of geniposide, magnolflorine, baicalin, berberine, oroxylin A­7­O­glucuronide, wogonoside, wogonin and oroxylin A in rat plasma (n=3)**

| **Components** | **Concentration (ng/mL)** | **Accuracy (%, Mean±SD)** | | | | | | |
| --- | --- | --- | --- | --- | --- | --- | --- | --- |
|  |  | **0 h at 4 ^◦^C** | **24 h at 4 ^◦^C** | **48 h at 4 ^◦^C** | **4 h at Room Temperature** | | **3 Freeze-thaw Cycles** | **15 Days Storage at -80 ^◦^C** |
| **Geniposide** | 22.00 | 91.41±2.46 | 92.45±3.77 | 94.47±7.50 | 96.79±9.73 | 103.98±13.49 | | 94.79±9.37 |
|  | 110.00 | 89.71±1.81 | 94.89±6.98 | 91.83±5.18 | 105.61±4.05 | 106.24±2.37 | | 93.47±2.81 |
|  | 1100.00 | 86.98±1.56 | 86.45±3.48 | 88.43±3.81 | 103.64±5.64 | 103.10±4.06 | | 84.24±4.45 |
| **Magnolflorine** | 0.44 | 97.50±1.43 | 113.55±2.72 | 94.68±6.31 | 92.23±4.63 | 98.36±12.10 | | 95.02±5.14 |
|  | 2.20 | 92.60±5.24 | 102.85±4.33 | 93.29±1.86 | 87.36±1.49 | 89.60±4.95 | | 97.62±5.20 |
|  | 22.00 | 103.60±2.43 | 107.98±0.35 | 101.93±1.06 | 99.85±2.93 | 99.31±1.00 | | 110.00±2.17 |
| **Baicalin** | 80.00 | 94.77±5.56 | 101.85±3.36 | 100.60±2.39 | 88.61±2.28 | 83.93±1.48 | | 108.37±3.48 |
|  | 400.00 | 88.16±1.30 | 87.69±1.91 | 87.53±0.55 | 88.74±7.14 | 85.60±1.39 | | 108.58±2.70 |
|  | 4000.00 | 113.21±2.34 | 113.82±1.03 | 114.55±2.43 | 91.88±1.25 | 89.57±1.01 | | 114.55±3.73 |
| **Berberine** | 0.03 | 99.40±1.13 | 89.91±3.63 | 92.33±6.28 | 91.06±5.85 | 86.90±3.03 | | 98.55±3.83 |
|  | 0.15 | 92.77±3.69 | 91.51±0.99 | 103.09±2.77 | 110.18±4.84 | 106.84±0.82 | | 108.90±6.26 |
|  | 1.50 | 89.08±3.01 | 91.71±1.03 | 97.23±0.72 | 101.33±1.95 | 105.88±5.34 | | 96.81±3.78 |
| **Oroxylin A-7-O-glucuronide** | 2.80 | 94.18±4.72 | 103.90±8.81 | 103.88±7.97 | 109.82±6.39 | 95.84±12.02 | | 107.28±4.05 |
|  | 14.00 | 95.64±3.48 | 87.28±5.05 | 90.71±3.84 | 84.65±2.37 | 87.55±6.21 | | 94.88±9.32 |
|  | 140.00 | 107.72±3.60 | 111.58±1.26 | 112.82±1.42 | 107.47±3.04 | 112.90±3.82 | | 111.52±2.77 |
| **Wogonoside** | 22.00 | 104.11±4.51 | 105.38±2.51 | 96.71±4.21 | 103.51±1.68 | 98.13±4.07 | | 104.20±3.97 |
|  | 110.00 | 86.10±1.66 | 86.22±2.85 | 89.35±0.70 | 82.81±2.44 | 82.86±4.03 | | 94.39±9.33 |
|  | 1100.00 | 109.90±3.35 | 113.20±1.08 | 114.80±1.92 | 95.31±3.50 | 90.40±3.10 | | 110.95±4.84 |
| **Wogonin** | 0.88 | 85.16±2.54 | 91.79±11.18 | 93.47±1.88 | 108.18±4.44 | 102.88±5.83 | | 81.22±1.01 |
|  | 4.40 | 91.13±1.57 | 89.76±5.00 | 94.57±1.43 | 91.43±6.85 | 96.49±6.68 | | 91.54±3.71 |
|  | 44.00 | 90.23±0.88 | 88.23±1.97 | 88.03±0.71 | 105.89±2.33 | 113.60±5.22 | | 91.90±3.42 |
| **Oroxylin A** | 0.54 | 98.26±3.48 | 101.06±3.86 | 106.07±5.41 | 106.28±7.77 | 102.24±5.35 | | 87.90±2.76 |
|  | 2.70 | 91.60±4.56 | 105.73±2.94 | 105.32±1.60 | 92.02±6.49 | 101.08±2.70 | | 88.92±0.08 |
|  | 27.00 | 94.92±2.22 | 104.92±3.38 | 108.80±2.64 | 110.34±1.63 | 112.02±3.89 | | 95.94±1.13 |
