# Supplementary material for: Pharmacokinetic-Pharmacodynamic Analysis on Inflammation Rat Model after Oral Administration of Huang Lian Jie Du Decoction
Source: PLoS One. 2016 Jun 9;11(6):e0156256. doi: 10.1371/journal.pone.0156256 (PMC4900566; doi:10.1371/journal.pone.0156256)
Supplement: S5 Table — (DOCX) [file pone.0156256.s007.docx]

**S5 Table. Extraction recovery and matrix effect of geniposide, magnolflorine, baicalin, berberine, oroxylin A­7­O­glucuronide, wogonoside, wogonin and oroxylin A in rat plasma (n=6)**

| **Components** | **Concentration (ng/mL)** | **Extraction Recovery (%, Mean±SD)** | **Matrix Effect (%, Mean±SD)** |
| --- | --- | --- | --- |
| **Geniposide** | 22.00 | 85.49±6.18 | 88.89±4.40 |
|  | 110.00 | 92.47±4.53 | 95.78±5.94 |
|  | 1100.00 | 85.02±2.40 | 89.75±2.83 |
| **Magnolflorine** | 0.44 | 92.20±3.22 | 93.88±3.54 |
|  | 2.20 | 94.35±7.00 | 93.39±7.59 |
|  | 22.00 | 87.60±4.62 | 88.05±2.80 |
| **Baicalin** | 80.00 | 68.52±3.78 | 75.73±5.42 |
|  | 400.00 | 80.20±2.74 | 70.80±3.68 |
|  | 4000.00 | 74.15±2.67 | 68.13±4.11 |
| **Berberine** | 0.03 | 95.79±4.96 | 114.50±6.94 |
|  | 0.15 | 95.66±3.69 | 103.70±3.89 |
|  | 1.50 | 85.82±2.64 | 104.96±4.72 |
| **Oroxylin A-7-O-glucuronide** | 2.80 | 104.50±9.08 | 94.90±9.07 |
|  | 14.00 | 96.42±9.16 | 87.05±7.68 |
|  | 140.00 | 91.18±7.68 | 82.92±2.90 |
| **Wogonoside** | 22.00 | 82.41±3.91 | 92.26±4.12 |
|  | 110.00 | 90.46±7.95 | 86.25±3.14 |
|  | 1100.00 | 82.48±3.39 | 82.27±4.04 |
| **Wogonin** | 0.88 | 99.30±6.55 | 85.64±4.77 |
|  | 4.40 | 105.98±6.35 | 85.75±5.76 |
|  | 44.00 | 98.33±5.91 | 85.95±4.98 |
| **Oroxylin A** | 0.54 | 89.22±6.49 | 81.22±4.91 |
|  | 2.70 | 94.83±5.56 | 83.74±3.74 |
|  | 27.00 | 85.69±2.68 | 92.97±4.97 |
